# New perspectives on the contribution of sanitary investments to mortality decline in English cities, 1845–1909

**DOI:** 10.1111/ehr.13195

**Published:** 2022-09-26

**Authors:** Toke S. Aidt, Romola J. Davenport, Felix Gray

**Affiliations:** ^1^ Faculty of Economics; ^2^ Department of Geography Cambridge Group for the History of Population and Social Structure

**Keywords:** Britain, faecal–oral diseases, mortality decline, public health, public investment, sanitation, urban infrastructure, water‐borne diseases

## Abstract

Health improved in English cities in the last third of the nineteenth century, in tandem with substantial increases in public spending on water supplies and sanitation. However, previous efforts to measure the contribution of public expenditures to mortality improvements have been hampered by difficulties in quantifying public health investments and the lack of mortality data for specifically urban populations. We improve upon the existing evidence base by (1) creating measures of the stock of urban district sanitary capital, by type, on the basis of capital expenditure flows, rather than loan stocks; (2) using mortality and capital stock data that relate to the same administrative units (urban districts), and (3) studying the period 1880–1909 as well as the earlier period from 1845. The stock of sewerage capital was robustly related to improvements in all‐cause mortality after 1880. The size of this effect varied with the extent of public investment in water supplies, suggesting complementarity between the two assets. For the period 1845–84, investments in water were associated with declines in infant and child mortality but the effect was much smaller and less precisely estimated in later decades. Our results suggest that improvements in water and sewerage targeted different transmission pathways for faecal–oral diseases.

One of the most protracted debates in British economic and demographic history concerns the relative contributions of economic growth and public health interventions to the early stages of the British mortality decline. McKeown and colleagues famously argued that improvements in living standards, particularly diet, substantially outweighed medical, public health, and other contributions before the twentieth century.^1^ While McKeown's critics have often argued that he under‐estimated the benefits of public health interventions, there remains no consensus on whether public investments in improving water quality and sewage disposal in the late nineteenth and early twentieth centuries had a major impact on mortality trends. Four recent papers have used broadly the same evidence for public investments and mortality rates to arrive at very different conclusions. Chapman argued that investments by urban sanitary authorities in public health initiatives, as measured by the stock of all loans (including some not related to public health), accounted for up to 60 per cent of the improvement in urban mortality rates between 1861 and 1900, and that reductions in the cost of borrowing after 1887 encouraged investment in water and sewerage, with falling infant mortality as consequence.^2^ Conversely, Hinde and Harris, in two related papers, used evidence of when loans were sanctioned and mortality for both ‘high‐performing’ towns and for all registration districts (RDs) over the longer period of 1851–1910 to argue that mortality fell almost everywhere and with similar patterns in rural and urban populations, regardless of wide variations in public investment in water supplies and sewerage.^3^

The historical study of public health and urban mortality patterns in England and Wales has been seriously hampered by the types of data available. The main problems are difficulties in quantifying public health infrastructure and the lack of mortality data for specifically urban populations. We partially overcome these problems using two novel sources: capital expenditures on water and sewerage by urban authorities, and weekly counts of deaths in the largest English towns. We improve upon the existing evidence base by (1) creating measures of the stock of municipal sanitary capital, by type, on the basis of capital expenditure flows, rather than debt stocks; (2) using mortality and capital stock data that relate to the same administrative units (urban districts), and (3) studying the period from 1880 to 1909 as well as the earlier period from 1845. The use of these sources came, however, at the price of sample size. Our main sample comprises only the 16 largest urban districts (excluding London) from 1880–1909 and is reduced to 11 when we exclude districts with private waterworks. Nonetheless, it includes roughly one‐quarter of the total urban population over the period.

Using these data aggregated to quinquennial intervals, we were able to estimate separately the contribution of water and sewerage capital stocks to all‐cause mortality decline between 1880 and 1909 and to investigate complementarity between the two types of public health capital. We found that the stock of sewerage capital was, holding the water capital constant, directly related to improvements in all‐cause mortality and could on its own account for about 13 per cent of the decline. Water capital, on the other hand, had an indirect effect in the sense that it interacted with the stock of sewer capital. Specifically, we found that the presence of water capital reinforced the capacity of sewerage capital to reduce mortality, suggesting complementarity between the two assets. These results pertain to the period when local government investments in public health boomed, following the Public Health Act of 1872. However, many towns developed waterworks and municipalised private waterworks in the decades before the 1870s (figure 1). To investigate whether these earlier investments in water supply were directly associated with improvements in mortality, we modelled this relationship for four towns for which we could construct a longer series of investments in water supplies. We found that the stock of water capital was negatively related to infant (and early childhood) mortality in the period 1845–84, but that the relationship had become much weaker by 1885. This is suggestive evidence that early water investments prior to the public health boom of the 1870s had important public health benefits.

**FIGURE 1:**
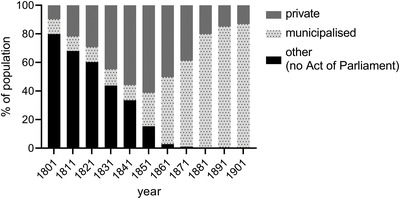
Percentage of the population served by municipal or private waterworks, or another source, 81 leading provincial towns and cities of England and Wales 1801–1901. *Note*: ‘Other’ includes towns with public water sources, such as some conduits, that were provided without an Act of Parliament, and towns with no ‘improved’ water supplies (relying wholly on river water, springs, rainwater, and wells). *Source*: Hassan, ‘Growth and impact’, table 3, p. 536

Section I reviews previous work in the area. Sections II and III, respectively, describe the construction of our measures of public health capital and mortality outcomes. Section IV presents panel regression analyses, and Section V concludes.

## I THE DEBATE

The most striking apparent demonstrations of the impact of improvements in water quality relate to the introduction of filtration and chlorination of public water supplies in American cities in the early twentieth century. These studies demonstrate very large reductions in typhoid mortality in response especially to water filtration.^4^ However, there is much less agreement over the impacts of water purification and sewerage treatment on other causes of death, with some studies reporting small or negligible reductions in infant and diarrhoeal mortality.^5^

In contrast to these findings for US cities, studies of water and sanitary improvements in nineteenth‐century cities elsewhere have generally reported modest effects on mortality. For example, fairly small or negligible beneficial effects of piped water supply and sewerage on infant mortality were reported for Munich and other urban districts in Bavaria from 1825–1909, for larger Finnish cities before *c*.1900, for Swedish urban centres from 1875–1930, for Swiss towns from 1876–1901, and for the Dutch town of Tilburg from 1904–6.^6^ However, larger reductions in infant mortality were observed in response to water and sewerage improvements in a larger sample of German towns from 1877–1913, in Sydney and in smaller Finnish towns in the early twentieth century, and the extension of modern sewerage was associated with large improvements in life expectancy in Parisian neighbourhoods from 1880–1914.^7^ As in the case of US cities, studies that considered typhoid generally observed much larger reductions in typhoid mortality compared with other causes of death, especially in response to improved water quality.^8^

Other authors have argued with evidence from Boston (1880–1920) and German cities (1877–1913) that interventions to prevent the faecal contamination of water (such as use of cleaner sources, filtration, and chlorination) are more effective when combined with modern sewerage to remove faecal waste.^9^ The importance of faecal disposal, as well as uncontaminated water, hints at the multiple pathways by which faecal‐oral diseases may be transmitted, which include fly‐borne transmission from exposed faeces to food and surfaces as well as faecal contamination of water.

**FIGURE 2:**
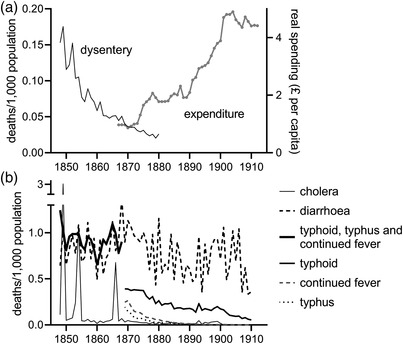
Crude death rates from faecal–oral diseases (1848–1911) and total local government expenditure (1868–1911), England and Wales. *Notes*: Expenditures are adjusted for inflation and population. *Sources*: *Local Taxation Returns*; Davenport, ‘Annual deaths’

In the British case there is also substantial disagreement about the efficacy of public health interventions to improve water supplies. English cities were some of the first in the world to introduce modern sewerage and clean piped water supplies. The first half of the nineteenth century was characterised by a major expansion in the provision of piped water, primarily by private water companies (figure 1). Hassan has argued that the short‐sighted nature of private investment proved detrimental to both industrial and public interests, and many towns therefore turned to public provision of water after mid‐century, building new municipal waterworks and acquiring private works.^10^ Some of the largest towns, including Liverpool and Manchester, undertook very ambitious long‐distance public supply schemes in the 1850s and 1860s. However, according to Hassan, the main beneficiaries of these early municipal waterworks were urban industries that required clean (and soft) water, and public health benefits were minimal.^11^ Only from the 1870s, he argues, did expenditures by local governments demonstrate a sustained and substantial rise, following the Public Health Acts of 1866, 1872, and 1875 which required towns to provide clean water and sewage disposal for their populations, and provided cheap finance to do so (figure 2a).^12^ Therefore, while early accounts of the public health movement in Britain attributed considerable success to the efforts of Edwin Chadwick and the Health of Towns movement in the 1840s, revisionist accounts now identify the 1870s as decisive.^13^

The strongest evidence for substantial effects of sanitary investments on public health in Britain has been provided by Chapman, who analysed the relationships between total outstanding debt contracted by urban districts and decadal mortality in English and Welsh towns. He restricted his analysis to the period 1861–1900, because data on the debt stock were not reported in the *Local Taxation Returns* before 1867, and because he considered that ‘this period marked the very beginning of the public health movement’.^14^ His decadal fixed effects panel regressions for a large sample of large and small urban districts indicated a strong negative relationship between total outstanding debt and crude death rates from both ‘airborne’ diseases and all causes between 1861 and 1900, and for ‘waterborne‘ diseases (cholera, typhoid, and diarrhoea) from 1871 to 1900.^15^ In a follow‐up paper, Chapman studies the link between cost of borrowing, investment in water and sewerage (as measured either by the flow of investment spending or as the stock of outstanding debt), and infant mortality for the same large sample of towns for the period 1887–1903 and finds that these investments reduced infant mortality substantially.^16^

Harris and Hinde used the same mortality data as Chapman, that is the Registrar‐General's decennial averages of deaths by age and cause for RDs. However, they used the full available data (1851–1910), for rural as well as urban RDs, and they used the information on age and cause of death to estimate the contribution of particular causes to improvements in life expectancy.^17^ Using a much longer and more comprehensive time series of loans which urban authorities sought approval for or contracted, and a more impressionistic analytical approach, Harris and Hinde found no obvious associations between loans sanctioned for water supplies or sanitary projects and improvements in mortality from ‘waterborne’ (a category that included typhus, in contrast to Chapman's) or other causes over the period 1851–1910.^18^ They noted that mortality fell in rural and urban districts regardless of sanitary investments and that typhus/typhoid and tuberculosis declined at similar rates in urban and rural areas.^19^

In a very insightful article, van Poppel and van der Heijden identified a number of conceptual and methodological difficulties involved in identifying relationships between water and sanitary interventions and mortality improvements.^20^ The most crucial issue is that while gut pathogens are often described as ‘water‐borne’, they can in fact infect humans via multiple routes. These diseases are described as ‘faecal–oral’ because they result from ingestion of infected faeces, and sources of infection include not only contaminated drinking water, but also poor personal hygiene, faecal contamination of crops and seafood, unsafe food preparation, transfer of faeces to food and skin by insects (especially flies), and even bathing in unclean water. Ewald demonstrated this variability in dependence on water‐borne transmission using evidence from historical outbreaks of faecal–oral diseases where the pathogen was identified and also the likely source of the infection. Ewald found that the most lethal faecal–oral diseases, including Asiatic cholera, typhoid, and dysentery, are the most dependent on water transmission, while those diarrhoeal pathogens that kill mainly very young children and the elderly are less commonly water‐borne and depend largely on human‐to‐human transmission (table 1).^21^ If this thesis is correct, then improvements in water quality could have had significant effects on those diseases that depended most on water‐borne transmission (cholera, typhoid, and dysentery). However, such improvements may have had little effect on diarrhoeal mortality. Conversely, diarrhoeal mortality may have been more sensitive to the removal of faecal waste from domestic or neighbourhood environments by sewerage systems. In the case of typhoid, by the late nineteenth century typhoid rates had converged in urban and rural populations in Britain, and there appears to have been a shift from predominantly water‐borne to food‐related epidemics associated especially with milk, ice cream, and shellfish, household transmission, and possibly with fly‐borne contamination.^22^

**TABLE 1:** Pathogens with faecal‐oral transmission routes

Pathogen	Mortality (% of cases)	Water‐borne outbreaks (% of all outbreaks)
*Vibrio cholerae*, classical biotype (Asiatic cholera)	15.7	83.3
*Shigella dysenteriae* type 1 (dysentery)	7.5	80
*Salmonella typhi* (typhoid)	5.8	74
*Vibrio cholera*, el tor biotype	1.44	50
*Shigella flexneri*	1.32	48.3
*Shigella sonnei*	0.65	27.8
Enterotoxic *E. coli*	<0.1	20
*Campylobacter jejuni*	<0.1	10.7
Non‐typhoid salmonella	<0.1	1.6

*Source*: Ewald, ‘Waterborne transmission’, pp. 83–119.

The multiplicity of potential influences on faecal–oral diseases is also suggested by the differences in trends in the major components of ‘water‐borne’ diseases (figure 2b). At the national level, cholera, one of the most water‐dependent diseases, virtually disappeared after 1866. Dysentery, also largely transmitted in water, had also declined to negligible levels by the 1870s. Typhoid, which was only reported separately from typhus (a louse‐borne disease with a very different aetiology) in 1869, showed a more complex two‐stage decline, with a period of major decline in the 1870s and early 1880s, followed by little further change until about 1900. Diarrhoeal diseases also followed a complex path, declining in the 1870s before rising again in the 1890s under the influence of a series of unusually hot summers, and then declining again in the first decade of the twentieth century. Most of the early decline in diarrhoeal deaths occurred amongst adults and older children (where water may have played a larger role of infection), with little improvement for infants before 1900.^23^ We consider these issues further in section III.

## II MEASURING THE STOCKS OF WATER AND SEWERAGE CAPITAL

Ideally, we want to measure the quantity and quality of the public health infrastructure in a town that is relevant to the mortality and life expectancy of the people living there over a given period. Unlike the American case, where improvements such as water filtration and sewerage were often relatively late and tended to affect most of a town's population fairly simultaneously, in Britain improvements in water supplies and sewerage occurred more gradually and accompanied the progressive extension of these facilities to households.^24^ Towns often relied on a variety of water sources, and initially filtered only the most polluted river water. Summer droughts also sometimes resulted in the substitution of inferior sources, provoking disease outbreaks in towns otherwise considered to have high quality water supplies. Critically for historians, the proportions of the population supplied with differing qualities of water and faecal disposal are poorly documented, and it is impossible to construct consistent time series of direct measures of water and sewerage provision and quality for most towns. Therefore, scholars have resorted to measures of investment in water and sewerage as proxies.

In his previous study of investment by English urban authorities in public health infrastructure, Chapman used total loans outstanding between 1867 and 1900 to approximate accumulated investment.^25^ There are three problems associated with this. First, these debts are not disaggregated by purpose, and therefore contain investment in a very wide variety of a town's activities, including activities such as municipal buildings, roads, markets, gasworks, public lighting, and trams, which have little to do with public health. Second, the debt at a given point in time depends on the repayment schedule of the underlying loans. That is, the stock can go up or down depending on when and how fast the loans are repaid. Third, the outstanding debt may include loans used for infrastructure that is no longer in use or loans that have been taken out but which have not yet resulted in operational capital.

To address these problems, we propose two alternative ways to use capital expenditure data from local government accounts and loans data from a range of archival and parliamentary sources to construct measures of the specific capital stocks associated with public health investments. In either case, we do this using the perpetual inventory method^26^ rather than relying solely on stocks of outstanding debt. The conceptual starting point is to write the evolution of sanitary capital (*S_it_*) per capita in town *i* in year *t* as

(1) $$\;S_{it}=S_{it-1}\;+I_{it}-\delta S_{it-1},$$

where *I_it_* is real per capita capital expenditures invested in the stock during the year, and *δ* is the depreciation rate. This approach avoids, with some caveats which we return to below, the three problems associated with the use of total debt stocks and generates yearly time series on the stock of sanitary capital by type in each town (which can then be aggregated to the quinquennial level). It is these stocks of cumulated investments, rather than the capital expenditures in a given year, which we conjecture will affect public health outcomes, likely with a lag.

We implement this general approach in two different ways using two data sources. The first source is the annual public accounts data from the *Local Taxation Returns* reported to Parliament. We refer to this as the *Local Taxation Returns* sample. These provide expenditure and debt data by town over the period 1867–1913.^27^ These data are available for many towns (approximately 1000), but we were constrained by the availability of mortality data recorded for the appropriate spatial units to study only the largest 16 cities,^28^ excluding London (see section III).^29^ In the *Local Taxation Returns*, data are reported at the geographical and administrative unit of the urban district.^30^ These were the local authorities tasked with managing and improving many aspects of urban life. They had the power to raise funds using local property taxes by running local natural monopolies (e.g. waterworks, gasworks, or markets), or taking out loans for investment purposes. The urban districts were responsible for spending on and investing in water and sewerage provision. Importantly, they could provide very little, and so minimise the local tax burden, or undertake expensive infrastructure projects if they perceived a benefit in doing so. From 1883, the *Local Taxation Returns* record the capital expenditure on water and sewers (‘expenditure defrayed out of loans’) annually. These expenditures represent what was spent on capital projects in the budget year as funds from loans sanctioned in the past were drawn down to fund investments in public health infrastructure. It is these expenditures that we use to construct the capital stocks from 1883 onwards. Before 1883, unfortunately, the accounts only record total (current and capital) expenditures, with expenditure on sewerage disaggregated from general public works from 1872, and in 1875 expenditure on water is likewise reported separately. We must decompose the total expenditures on water and sewerage into current and capital expenditures in order to get an estimate of *I_it_* for this period. We do this by estimating the urban district‐specific relationship between capital expenditures (*I_it_*) and total spending for a period of years in which we have disaggregated data and using that to back out capital expenditures before 1883. We detail in supplementary appendix A how this is done. This gives us a panel of water (1875–1910) and sewerage capital expenditures (1872–1910) from which we can construct the two public health capital stock variables. To apply the perpetual inventory method to construct these capital stocks we need to pin down the initial stocks, but we lack such data. Instead, we use the value of loans outstanding in 1884, the first year in which it is reported separately for water and sewers, or the first year after municipalisation of water.^31^ For the depreciation rate *δ*, we assume a value of zero in the baseline, with the rationale that *current* spending included maintenance of the capital stock. It makes no substantial difference to our statistical analysis whether we apply a positive value for *δ* to take into account that the stocks built with capital expenditures in the far past may no longer exist.^32^

The second source is the data that were created by Harris and Hinde.^33^ They painstakingly collected data, from a range of archival and parliamentary sources, on total loans sanctioned by central government, and divided these loans by purpose, separating loans relating to water supplies from loans for other health‐related purposes and functions. These data give the values of the loans sanctioned in each year by the Local Government Board under the various Public Health and Local Confirmation Acts, by Local Acts of Parliament, or by the General Board of Health and the Privy Council for water supply. Accordingly, the raw data are loans sanctioned in a given year, rather than expenditures defrayed from loans taken out in the past during that year. We use these data to proxy *I_it_* in equation 1 and calculate the stock of water capital for the period 1817–1909. In doing so, we assume that the initial stock in 1817 is the same in all towns and normalise it to zero.^34^ The great advantage of this source is that the data can be traced back to when the earliest mortality data (1845) are available (see section III). The downsides are that the data are only readily available for four towns, that we can only construct estimates of the water capital stock, and that the construction is based on when a loan was sanctioned rather than when the funds made available from the loan were actually spent on water infrastructure.^35^ The last issue is likely to introduce complicated lags in the mapping from the stocks to public health outcomes as it took time before the funds sanctioned by a loan got converted into water infrastructure, and we try to take this into account in the statistical analysis.^36^ We refer to this sample as the *Loans* sample. The capital stocks constructed from the *Loans* or the *Local Taxation Returns* samples are converted into real 1911 pounds using the Rousseaux price index. For the statistical analysis, all data are aggregated to five‐year (quinquennial) periods. This removes minor year‐on‐year variation in the stocks and isolates periods with major investments.

**FIGURE 3:**
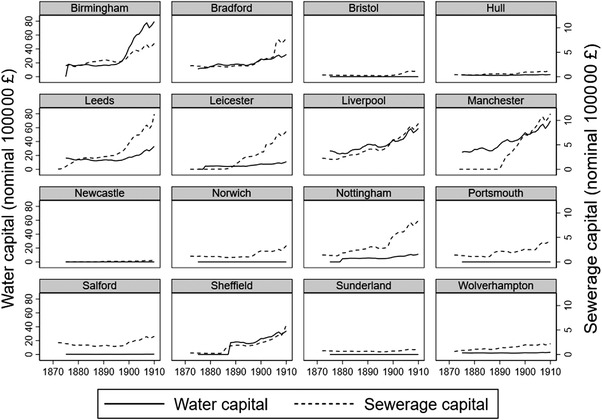
Sewerage and water capital in the 16 urban districts in the *Local Taxation Returns* sample, 1872–1910. *Notes*: The sewerage and water capital stocks are recorded in 100 000 nominal pounds. Bristol, Newcastle, Norwich, Portsmouth, and Sunderland had private waterworks throughout the period, and Birmingham (municipalised in 1876), Leicester (in 1878), Nottingham (in 1880), and Sheffield (in 1887). *Source*: *Local Taxation Returns*

**FIGURE 4:**
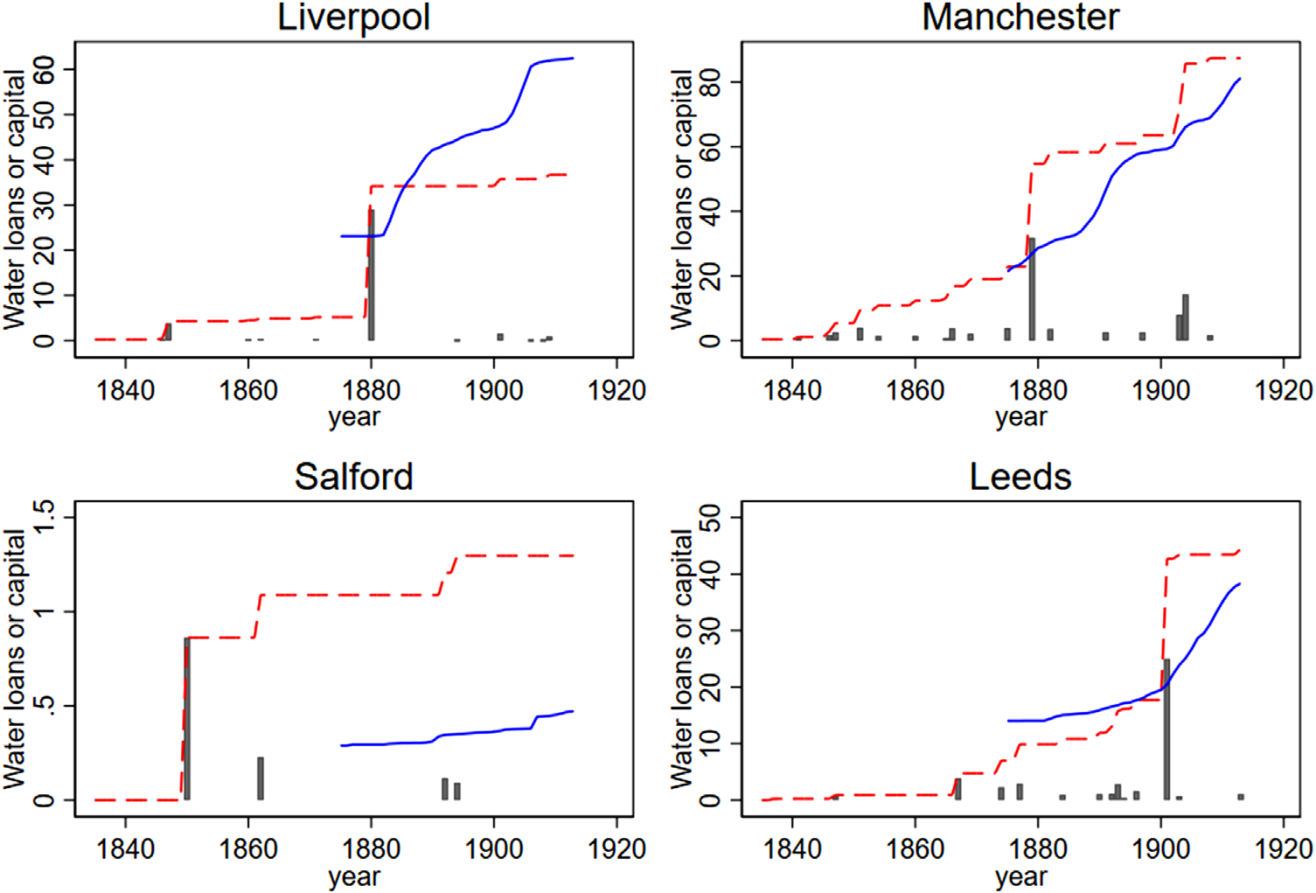
Water loans and associated water capital data for the four towns in the *Loans* sample between 1835 and 1910. *Notes*: The grey bars represent the loans sanctioned, the dotted line is water capital (measured in 100 000 nominal pounds) from Harris and Hinde, and the solid line is water capital (measured in 100 000 nominal pounds) from the *Local Taxation Returns*. *Sources*: Harris and Hinde, ‘Sanitary investment’, *Local Taxation Returns* [Colour figure can be viewed at wileyonlinelibrary.com]

Figure 3 shows water and sewerage capital for each of the 16 urban districts by year from 1872 to 1910 in the *Local Taxation Returns* sample, and figure 4 shows the water loans and the associated water capital data for the four towns in the *Loans* sample between 1835 and 1910. Figure 3 reveals wide variations in the stock of water and sewerage capital over time and between urban districts, especially in the provision of water infrastructure. Some of this variation was caused by reliance on private waterworks. Of the 16 urban districts, 9 had private waterworks in 1870, and in 5 districts water supplies remained in private ownership in 1911 (Bristol, Newcastle, Norwich, Portsmouth, and Sunderland). These five urban districts were, unsurprisingly, characterised by very low public investments in water and the recorded stock of water capital is very small. Four urban districts in our sample municipalised existing private waterworks in the period under study. Municipalisation was associated with a sudden rise in public water capital expenditures in Birmingham (municipalised in 1876), Leicester (1878), Nottingham (1880), and Sheffield (1887). Importantly, these very substantial public investments may have had no immediate effect on the quality or extent of the water supply, because the money was used in the first instance to acquire existing waterworks. Moreover, municipalisation was often triggered by the need to expand existing provision, and so further capital expenditure was often required simply to maintain per capita supply in the face of urban growth.^37^ In the statistical analysis, we take this into account by restricting the sample to urban district–year pairs for which water was municipalised. This eliminates the five towns with private waterworks throughout and thus reduces the sample to 11 districts and the years for which the districts that eventually municipalised had public water supply. For towns with public water supplies, investments after the initial building or acquisition of a waterworks represented a variety of activities. Some were incremental in the sense that they involved progressive improvements to existing supplies (such as filtration of the more polluted sources, increases in reservoir capacities, or the extension of in‐house supplies to more households). Others represented very substantial investments in new sources. Birmingham, for example, relied on access to deep local aquifers until the end of the nineteenth century, when the urban district was finally forced to establish a long‐distance water supply from Wales. However, even such a major investment did not necessarily result in any *improvement* in water quality (which was already high), but pre‐empted a deterioration in supply in the face of continued urban expansion. Our results tentatively suggest that with respect to water supplies, the largest improvements in mortality were associated with early investments to establish a relatively clean water supply, and later investments faced diminishing returns.

Figure 4 shows investment in water in Liverpool, Manchester, Salford, and Leeds from 1835 to 1910. These four towns municipalised water in 1848, 1851, 1850, and 1852, respectively. The bars indicate the size of loans taken out in different years, while the dotted line shows the accumulation of water capital stocks. The solid line shows water capital constructed from the *Local Taxation Returns* for comparison of the trends. Again, there were large differences across time and space in when loans were taken out to fund water works and in their magnitude. Unlike the *Local Taxation Returns* sample, the *Loans* sample includes Private Acts loans taken out by private companies and in the statistical analysis we utilise water stock data from 1840 in the baseline. This includes years with private water supply but we show that our results are not sensitive to that.^38^

Some of the variation evident in figures 3 and 4 also reflects the fact that many urban districts sold water to neighbouring rural and urban districts.^39^ Manchester, for example, invested in very expensive long‐distance water supplies, constructing reservoirs and transporting clean upland water from Longendale in the Pennines in the 1850s and from Thirlmere, over 90 miles away in the Lake District, in the 1890s. The water was then supplied both to Manchester residents and to surrounding districts, including Salford. Therefore, in our data the urban district of Salford invested little in water yet benefitted from the investments made by Manchester. We investigate how this influences our results.

Investments in sewerage were more modest, and the stocks generally demonstrated smoother rises across the period 1872 to 1910 for which we have data on sewerage capital (figure 3). Early attempts to privatise sewerage and faecal collection failed, and all town councils had to deal with their own local wastes. However, the methods employed varied.^40^ Flush toilets were still not universal by 1911. Many urban districts focused in the early part of our period on replacing the most egregious hazards, such as midden privies (where faecal waste was stored for months in pits near houses), with other dry conservancy methods (involving storage of faecal waste in removable pans or pails that were emptied and replaced regularly). This type of public health expenditure is not captured by our sewerage capital stock. Instead, sewerage investments were in the provision of sewers for faecal waste from water‐flushed toilets, and in sewage treatment for the chemical and mechanical breakdown of faecal waste to destroy pathogens. These elements were not provided in a synchronised fashion. Most water‐borne sewage was initially dumped, untreated, into waterways. The recognition that faecal contamination of drinking water was dangerous initially resulted only in the separation of contaminated and uncontaminated water, as sewage outfalls were removed further downstream, and water supplies either filtered or sourced from more remote areas. The purification of sewage was costly and was not necessarily of direct benefit to urban districts, because the main advantages often accrued to downstream users. Clear guidelines for chemical and mechanical treatment of sewage were only developed after 1898.^41^ Unfortunately, the *Local Taxation Returns* do not unpick the components of investments in sewage disposal versus treatment.

## III THE *WEEKLY RETURNS* AND MORTALITY AT THE URBAN DISTRICT LEVEL

The study of urban mortality patterns in Victorian England and Wales has been severely hampered by the reporting practices of the Registrar‐General's office. The two most serious problems are the geographical units for which most mortality data were reported before 1912, and the ways that deaths by individual causes were aggregated and reported. Our study largely overcame the first problem (for the period 1875–1910) but not the second.

**FIGURE 5:**
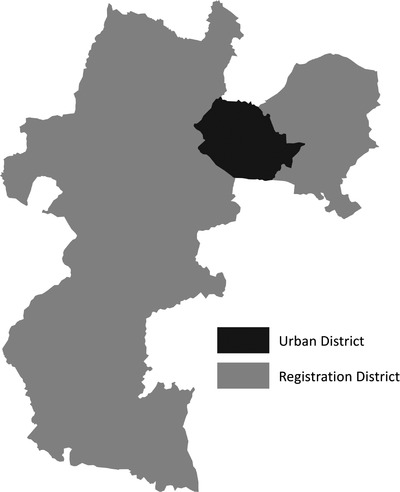
Wolverhampton urban district and registration district, 1911. *Source*: Day, ‘Registration sub‐district boundaries’; Satchell et al., ‘Selected urban districts’

The main administrative unit used by the Registrar‐General to report deaths before 1912 was the RD. These were based on poor law unions created from the aggregation of groups of parishes under the Poor Law Amendment Act of 1834. The unions were often designed to combine rural with urban populations to spread the costs of supporting the urban poor. Thus, except in the case of some districts in the largest towns, RDs were generally larger than their constituent towns in area and population, and included a mix of rural and urban communities. Public health investments, on the other hand, were undertaken by urban districts (section II). Urban districts were often coterminous with existing borough boundaries, and were intended to represent mainly urban populations. The reporting of deaths by RD, therefore, poses a potentially serious problem for studies of the health effects of urban investments in water supply and sewerage systems, because investments made by urban districts would affect only that fraction of the RD populations that lived within the urban district.^42^ Figure 5 illustrates the point for Wolverhampton, which represents the situation that was typical of many smaller towns (but not of many of the large urban districts in our sample; see supplementary appendix table A1).

To overcome the mismatch problem, we use a previously unexploited source of mortality data that pertains to urban districts, not RDs. This is the *Weekly Returns of the Registrar‐General*, which reported deaths from a limited set of causes for the largest urban districts from 1870 to 1911. The series started in 1870 with the 16 urban districts (and London) with populations that approached or surpassed 100 000 in 1861.^43^ While the 16 urban districts constitute a small panel compared with the roughly 1000 urban districts in England and Wales in this period, they accounted for around a quarter of the total population of non‐metropolitan urban districts, and almost all of the population living in large provincial towns (with populations of 100 000 or more; see supplementary appendix table A1).

A central issue is which mortality rates provide the best measure of the success of sanitary interventions to reduce faecal exposure. As we argued in section I, diseases traditionally classified by historians as ‘water‐borne’ differed markedly in their dependence on contaminated water (table 1). This is particularly problematic in the English case because the Registrar‐General did not provide a full breakdown of deaths by individual causes at the sub‐national level, making it difficult to track trends in individual faecal–oral diseases over time.

Previous studies have used as outcome measures all‐cause, typhoid, infant, or diarrhoeal mortality rates, or aggregated categories of ‘water‐borne’ causes of death that either included or excluded typhus (table 2). The *Weekly Returns* reported weekly counts of total births and deaths, deaths aged under one year and 60 years and over, and deaths attributed to a limited number of causes, including diarrhoea, cholera, fever (which included deaths from typhus, typhoid, and ‘simple continued fever’), and several other infectious diseases. We used these to construct all‐cause, infant, and diarrhoeal mortality rates as our main outcome measures for the period 1875–1910. We could not construct a broad ‘water‐borne’ disease category, because the *Weekly Returns* did not distinguish typhoid from other ill‐defined ‘fevers’, and dysentery was not reported – but was anyway a very minor cause of death by 1870, as was cholera (figure 2). Diarrhoea, however, accounted for around 80 per cent of all deaths attributed to ‘water‐borne’ (or faecal–oral) diseases by 1875 and was a major component of the ‘urban penalty’ (figure 2). Nonetheless, typhoid appears to have provided a much more sensitive indicator of water quality in previous studies, and it was unfortunate that it was not reported separately in our source. To provide a partial remedy subject to the mismatch problem, we constructed typhoid mortality rates for the central RDs corresponding to our 16 urban districts (table 2).

**TABLE 2:** The main sources of mortality data reported at sub‐national level by the Registrar‐General (RG) between 1850 and 1911

Source	Period	Spatial units	Frequency	Ages	Faecal‐oral diseases	Previous uses	This study
Decennial returns	1851–1910	Registration districts	Decadal averages	Yes	Yes^a^	Chapman; Hinde & Harris; Woods	All‐cause, 1861–1910
Annual returns	1838–84	Registration districts	Annual	Yes	No		All‐cause and infant, 1845–84
Annual returns	1856–1911	Registration districts	Annual	No	Yes	Beach et al.	Typhoid and typhus, 1869–1911
Quarterly returns	1870–1911	Registration sub‐districts	Three monthly	No	Some^b^	Chapman	Infant, 1885–1909
Weekly returns	1870–1911	Selected urban districts	Weekly	No	Some^b^		All‐cause, infant, and diarrhoea, 1880–1909

*Sources*: *8th–74th Annual Reports of the Registrar‐General*; Beach et al., ‘Urban water systems’; Chapman, ‘Contribution of infrastructure’; idem, ‘Interest rates’; Hinde & Harris, ‘Mortality decline’; *Quarterly Returns*; *Weekly Returns*; Woods, ‘Causes of death’; idem, *Demography*.

^a^Chapman's ‘water‐borne’ category included typhoid, cholera, diarrhoea, and dysentery (1871–1900) (Chapman, ‘Contribution of infrastructure’). Hinde and Harris's ‘water‐borne’ category included typhus as well as typhoid, cholera, diarrhoea, and dysentery (1851–1910).

^b^Diarrhoeal and cholera deaths.

We aggregated weekly deaths from the *Weekly Returns* into annual and then quinquennial series to match our constructed water and sewerage capital data in the *Local Taxation Returns* sample, and converted these to mortality rates using annual population estimates interpolated geometrically from decennial census reports.^44^ The resulting death rates were crude rates and were calculated using the whole population as the denominator. For infant and diarrhoeal deaths, we constructed rates per 1000 births, because most or all of the deaths in these categories occurred to infants. For comparison with other studies, we also constructed crude all‐cause decennial death rates, and annual and quinquennial crude death rates for typhoid and typhus for the central RDs associated with our 16 urban districts.

To trace the impacts of earlier investments in water recorded in the *Loans* sample on mortality, we constructed annual and quinquennial mortality rates for Leeds, Liverpool, Manchester, and Salford for 1845–84. The Registrar‐General did not report deaths by cause in RDs before 1856. However, between 1838 and 1884 deaths were reported (from all causes) by age groups for individual RDs, allowing us to construct life expectancy measures for the central RDs of the four towns for the years 1845–84.^45^ Although the Registrar‐General discontinued the reporting of deaths by age in RDs after 1884, infant deaths continued to be reported (in the *Quarterly Returns*), and so our measure of infant mortality extends across the whole period 1845–1911. Annual counts of deaths by age groups were converted into age‐specific mortality rates using annual population estimates interpolated geometrically (for each age group) from decennial census reports.^46^ Life expectancy was calculated using abridged life tables for age groups 0, 1–4, 5–9, 10–14, and then 10‐year age groups to 85. Life years remaining in the last (open) interval were calculated as equal to the number alive divided by the age‐specific death rate.

**FIGURE 6:**
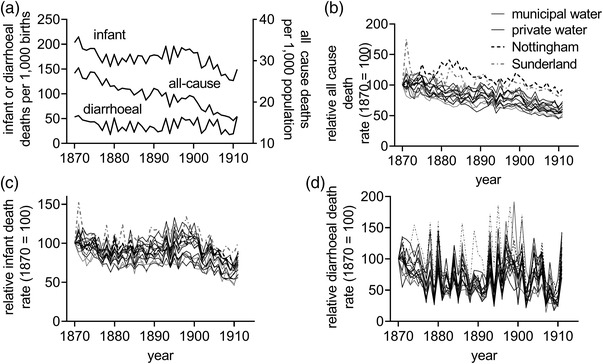
All‐cause, infant, and diarrhoeal mortality rates in 16 panel urban districts, 1870–1911. *Notes*: Panel a represents aggregated rates for all 16 urban districts (weighted by population). Panels b–d represent rates standardised to the rate in 1870. All‐cause mortality rates are total annual deaths per 1000 population, infant and diarrhoeal mortality rates are per 1000 births. *Source*: *Weekly Returns*

Figure 6 displays trends in the mortality measures from the *Weekly Returns* from 1870 as average rates for the urban districts in the *Local Taxation Returns* sample as a whole (figure 6a) and normalised to 1870 for each town (figure 6b–d). In aggregate, crude all‐cause death rates declined fairly continuously between 1870 and 1911 (figure 6a). This was also the case in individual urban districts, with the exception of two very poorly performing districts, Nottingham and Sutherland (figure 6b). In contrast to all‐cause mortality, our two more specific measures of sanitary conditions, diarrhoeal and infant mortality, showed little improvement before *c*.1901, and actually rose in the 1890s (figures 6a,c,d). This pattern reflected national trends and was attributed to a string of hot, dry summers in the second half of the 1890s that caused major summer diarrhoeal epidemics.^47^ Indeed, figure 6d draws attention to the remarkable synchrony of inter‐annual variations in diarrhoeal mortality across all urban districts, regardless of the differences in *levels* of mortality.

**FIGURE 7:**
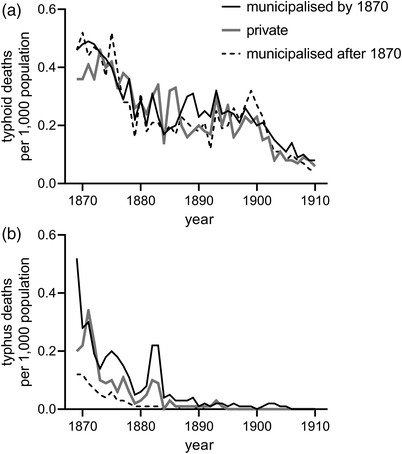
Typhoid (a) an typhus (b) mortality rates in registration districts (RD) associated with our 16 panel urban districts, by type of ownership of water supply. *Note*s: Typhus and typhoid were only reported separately in annual reports from 1869. Rates are per 1000 population. *Source*: *32nd–74th Annual Reports of the Registrar‐General*

We could not measure typhoid mortality in the 16 urban districts, however, death rates from this disease fell markedly in the period 1869–80 in the associated RDs (figure 7a). There was, however, no further improvement in typhoid mortality until *c*.1900, despite large increases in investment in water and sewers, and there were no obvious differences in levels or trends in mortality from these diseases in towns with municipalised water compared with those with private supplies. Trends in typhoid mortality clearly differed from those of diarrhoeal mortality, a fact suggestive of different aetiological pathways (section I). Louse‐borne typhus displayed different geographical and chronological patterns compared with typhoid, and had virtually disappeared as a cause of death by 1890 (figure 7b).

**FIGURE 8:**
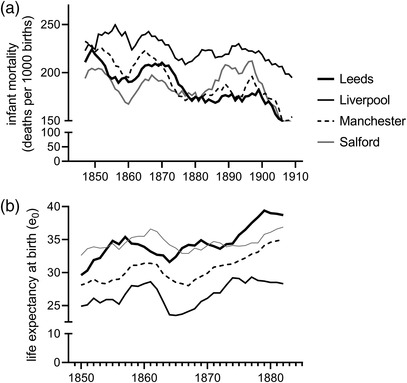
Life expectancy at birth and infant mortality, four towns. *Notes*: Rates are five‐year moving averages. A single eponymous RD was used in each case except Leeds (which was reported with Hunslett in 1845–6) and Manchester, which was split into Manchester and Prestwich RDs in 1874. For Leeds, rates for 1845–6 were calculated using Leeds’ intercensal population and reduced by 100/190 to reflect the relative populations of Leeds and Hunslet RDs in 1841 and 1851. For Manchester, rates include Prestwich RD (Prestwich is also included in the fiscal data for Manchester). The censuses for 1851–81 did not report age groups separately for ages under five years. To estimate the population aged 1–4, births in the census year were subtracted from the population aged 0–4, and deaths under 1 were added. *Sources*: *8th–47th Annual Reports of the Registrar‐General*; *Quarterly Returns of Marriages, Births and Deaths*, 1885–1911; Census of Great Britain, 1851; Censuses of England and Wales, 1861–1911

Figure 8 displays trends in infant mortality (1845–1911) and life expectancy (1845–84) in the four towns in our *Loans* sample (rates are five‐year moving averages to display trends more clearly). Infant mortality fell markedly before 1880 and again after 1900, in line with national trends (see figure 6a,c). Life expectancy dipped in the Lancashire towns (Liverpool, Manchester, and Salford) during the ‘cotton famine’ of the early 1860s (in contrast to trends in infant mortality), before rising in the 1870s.^48^

## IV EMPIRICAL SPECIFICATION AND RESULTS

Table 3 provides descriptive statistics for the main data used in the statistical analysis. The *Local Taxation Returns* sample is an unbalanced panel with information on the urban district‐quinquennial observations with municipalised water supply between 1880 and 1909.^49^ This covers the seven urban districts that had municipalised water supply for all the years and the four that had for some (*N* = 11). The length of the panel is constrained by the availability of water capital data (1875) and the quinquennial period ending in 1909 is the last with data for five years. To make use of the entire sample, we also report some specifications with data from all 16 urban districts. While sewerage treatment was under municipal control everywhere, this sample includes periods with private waterworks. The three advantages of this dataset are that we can measure water and sewerage capital separately, that all mortality data are recorded for the correct geographical units, and that the capital stocks are constructed from actual capital expenditure rather than from loans sanctioned. A drawback is that we cannot study the period before 1880 (i.e. in our lagged models, we use capital data for the period 1875–1904, and mortality data for the period 1880–1909). The *Loans* sample goes back to 1845, which is the first full five‐year period with mortality and life expectancy data (the loans data go back to 1817 and there exists information on the average infant mortality for 1838–44). The drawbacks of using this sample are that the mortality data are recorded at the RD level; that a consistent time series based on yearly data cannot, except for the infant mortality rate, be constructed beyond 1884; that the stocks are constructed from loans sanctioned, which does not in general correspond to the time when the funds were spent on water capital projects; and, most importantly, that we only have data on loans raised for water supply for four towns (*N* = 4).

**TABLE 3:** Descriptive statistics

	Unit	N	Mean	SD	Min	Max
***Local Taxation Returns* sample (1870–1909)**						
*Weekly returns of the RG*						
All‐cause mortality rate	UD	81	21.6	3.86	13.7	30.5
Diarrhoea mortality rate	UD	81	38.6	8.76	23.5	58.9
Infant mortality rate	UD	81	176	19.5	131	239
Crude birth rate	UD	81	34.0	4.74	20.3	44.9
*Annual returns of the RG*						
Typhus mortality rate	RD	81	0.033	0.076	0	0.39
Typhoid mortality rate	RD	81	0.24	0.12	0.017	0.68
*Local Taxation Returns*						
Water capital^a^	UD	74	5.16	3.43	0.16	15.6
Sewerage capital	UD	81	1.12	0.77	0	3.45
Debt outstanding	UD	81	14.5	8.21	0.56	33.5
Tax base	UD	81	4.63	1.34	2.06	7.38
*Census*						
Population growth	UD	81	0.0149	0.0094	−0.0064	0.0392
Population	UD	81	300 177	163 308	69 010	724 471
Female	UD	81	0.52	0.0097	0.50	0.54
Aged 0–14	UD	81	0.35	0.025	0.27	0.39
Aged 15–44	UD	81	0.48	0.017	0.43	0.51
Manufacturing employment	UD	81	0.10	0.070	0.036	0.24
Textiles employment	UD	81	0.092	0.10	0.0028	0.35
*Other*						
Municipalised water^b^	UD	128	0.62	0.48	0	1
***Loans* sample (1845–1909)**						
*Annual returns of the RG*						
Infant mortality rate	RD	52	198	24.2	139	243
Early childhood mortality rate	RD	32	226	47.8	145	357
Crude death rate	RD	52	28.5	5.33	17.4	42.5
Life expectancy at birth	RD	32	31.3	4.08	22.5	38.7
Life expectancy at 15 years	RD	32	38.2	2.55	32.8	42.3
Crude birth rate	RD	52	36.1	3.61	26.3	44.9
*Harris and Hinde*						
Water capital per 1000 cap.	UD	52	4.31	4.95	0	16.4
Water capital per 1000 cap. (3%)	UD	52	2.80	3.16	0	12.7
*Census*						
Population growth (%)	UD	52	1.64	1.05	−0.65	4.70
Population	UD	52	342 513	167 342	59 375	721 254

*Notes*: The data refer to five‐year averages and we report data, if available, from 1870–4 to 1905–9 for the 11 urban districts in the core *Local Taxation Returns* sample with municipalised waterworks (seven districts had municipalised water from 1870, two from 1875–9, one from 1880–4, and one from 1885–90) and from 1845–9 to 1905–9 for the four urban districts in the *Loans* sample. Not all variables are available for the full periods for the two samples: column *N* gives the number of observations for each variable. Crude birth and all‐cause and typhoid and typhus death rates are per 1000 population, while diarrhoeal and infant mortality are per 1000 births. Early childhood mortality rate is the probability of dying between exact ages one and five years, per 1000 children surviving to exact age one. All variables derived from the *Local Taxation Returns* are in (real) £ per person, the water capital variables in the *Loans* sample are in (real) £ per person (without depreciation and with 3% depreciation). Census data are exponential interpolations of decadal data. Sex and age variables are normalised by the total population. Employment variables are normalised by the working age population (15–64). RG, Registrar‐General; UD, urban district; RD, registration district.

*Sources*: *8th–47th Annual Reports of the Registrar‐General*; Census of Great Britain, 1851; Censuses of England and Wales 1861–1911; Harris and Hinde (unpubl. data); *Local Taxation Returns* 1870–1909; *Quarterly Returns of Marriages, Births and Deaths*, 1885–1911; *Weekly Returns* 1870‐1909.

^a^ There are only 74 district/period observations for water capital because water expenses were not recorded before 1875.

^b^ Municipal records the fraction of a five‐year period that a district had municipalised water and refers to the full sample of 16 districts for eight periods.

To quantify the relationship between water and sewerage capital and mortality, we follow Chapman^50^ and estimate a two‐way fixed effects panel regression model. For the *Local Taxation Returns* sample, the model can be written as

(2) $$\;M_{it}=\;\alpha +\beta_{1}WC_{it-1}+\beta_{2}SC_{it-1}+\gamma X_{it}+\eta_{i}+\lambda_{t}+\varepsilon_{it},$$

where *M_it_* is a mortality rate for urban district *i* in five‐year period *t*, *WC_it‐1_* and *SC_it‐1_* are water capital and sewerage capital (real per capita) in period *t‐1*, *X_it_* is a vector of controls, *η_i_* is an urban district fixed effect, *λ_t_* is a time effect, and *ε_it_* is the residual. In some specifications, we include the interaction between the two capital stocks to allow the effects to be conditional and to test for complementarity between water and sewerage capital. The two‐way fixed effects estimator removes time‐invariant and urban district‐invariant confounders in the relationship between sanitation capital and mortality. To reduce the risk of reverse causality and to allow sanitation capital time to affect mortality, we estimate equation 2 with the first lag of *WC_it‐1_* and *SC_it‐1_* and thus model mortality from 1880 onwards. The specification for the *Loans* sample is similar, except that we only have information on water capital (real per capita), that the set of control variables is more limited, and that the mortality outcome variables are measured at the RD level. In the results reported below, the coefficients are standardised and the regressions are weighted by average population over the period, so that larger urban districts have a proportionately larger influence on the coefficients. The importance of clustering the standard errors at the urban district level in fixed effects panel models is well known,^51^ but we have few districts (4–16 depending on the sample), so it is possible that the standard errors calculated from the cluster‐robust covariance matrix for fixed effects models^52^ are insufficiently conservative.^53^ We therefore also report clustered standard errors (*p*‐values) on the basis of a wild bootstrap procedure.^54^

Table 4 reports the results of estimating equation 2 on the *Local Taxation Returns* sample, with all‐cause mortality as the outcome variable and the lag of water and sewerage capital as the measures of sanitation infrastructure. The three first columns build up the model to the full specification with all fixed effects and controls. Column (1) shows the raw correlation. The *R* ^2^ suggests that the two sanitation variables alone can explain about 35 per cent of the variation. Both point estimates are negative, but only the estimate for sewerage capital is statistically significant and this is true irrespective of whether the standard errors are calculated from the cluster‐robust covariance matrix (reported under the coefficient estimates) or with a wild bootstrap, as indicated with the *p*‐values at the bottom of the table. Columns (2) and (3) include the full set of time‐varying controls (tax base, population growth, female, aged 0–14, aged 15–44, birth rate, manufacturing employment, and textiles employment) and progressively add urban district and time fixed effects.^55^ We see that the (within) *R* ^2^ increases to 88–96 per cent with most of the additional explanatory power coming from the controls and the time fixed effects. Sewerage capital remains significant, but the effect size is three times smaller in the complete specification in column (3). The coefficient on the water capital variable is about 59 per cent smaller and insignificant on its own, but we note that the two coefficients are jointly significant. To illustrate the effect size, we report at the bottom of the table the percentage of the observed decline in mortality that can be explained by the point estimate of the two sanitation variables, respectively.^56^ Sewerage capital can explain about 13 per cent of the decline in all‐cause mortality between 1880 and 1909, and if we add the effect of water capital, the decline explained increases to 17 per cent. This is substantial, but also leaves room for many other factors to have contributed.

**TABLE 4:** All‐cause mortality (1880–1909) and water and sewerage capital: regressions results from the *Local Taxation Returns* sample

	All‐cause mortality rate per 1000 population						
Dependent variable	(1)	(2)	(3)	(4)	(5)	(6)	(7)
Water capital (WC) t‐1	−0.0099 (−0.058)	−0.0019 (−0.018)	−0.087 (−1.17)	0.055 (0.85)	−0.058 (−0.85)	0.023 (0.29)	−0.37* (−1.90)
Sewerage capital (SC) t‐1	−0.72*** (−3.98)	−0.40** (−2.42)	−0.21** (−2.85)	−0.16*** (−6.05)	−0.18** (−2.15)	−0.060 (−1.10)	−0.34*** (−2.81)
WC × SC interaction t‐1				−0.28** (−2.62)		−0.23*** (−3.80)	
Municipalised water					0.10 (1.25)	−0.06 (−0.99)	
Observations	63	63	63	63	96	96	52
*R* ^2^	0.347	0.877	0.957	0.975	0.946	0.963	0.962
Town fixed effects (FE)	NO	YES	YES	YES	YES	YES	YES
Time FE	NO	NO	YES	YES	YES	YES	YES
Controls	NO	YES	YES	YES	YES	YES	YES
Method	OLS	OLS	OLS	OLS	OLS	OLS	LIML
Period	1880–1909	1880–1909	1880–1909	1880–1909	1880–1909	1880–1909	1885–1909
Standard errors	Clustered	Clustered	Clustered	Clustered	Clustered	Clustered	Robust
*p*‐value (water)^a^	0.95	0.98	0.22	0.53	0.40	0.82	0.22
*p*‐value (sewerage)^a^	0.021	0.17	0.026	0.0090	0.10	0.32	0.048
*p*‐value (joint)^a^	0.046	0.24	0.037	0.024	0.21	0.0081	0.096
*p*‐value (inter)^a^				0.21		0.093	
Decline explained (W)^b^	0.46	0.090	4.00		2.67		17.1
Decline explained (S)^b^	45.7	25.2	13.3		11.7		22.0
Towns	11	11	11	11	16	16	11
K–P *F*‐statistic^c^							4.75
Selection ratio^d^			1.11		0.97		

*Notes*: Data for water and sewerage capital pertain to 1875–1909 but as we lag these two variables, we model mortality over the period 1880–1909. The data are aggregated to five‐year periods; we keep the four towns that municipalised water after 1875 in the sample if water supply was municipalised in part of a five‐year period (but take the average of the two capital stocks over the post‐municipalisation years only). All coefficients are standardised. Standard errors are clustered at the town level in columns (1) to (6); *t*‐statistics given in parentheses. *R* ^2^ calculated for within variation when specification includes fixed effects. Regressions are weighted by average population over the period. All variables are measured at the urban district level. Control variables included in all regressions except column (1) are tax base, population growth, female, aged 0–14, aged 15–44, birth rate, manufacturing employment, and textiles employment. Columns (1)–(4) and (7) include town‐year observations where a town's water supply is municipally owned (and its expenditure therefore appears in its public accounts). Columns (5) and (6) are for the full balanced panel of all towns and include a control for whether water supply is municipalised or private. Column (7) reports an IV specification. Estimated with LIML, where water and sewerage capital (lagged) is instrumented by its second lag. ***p < 0.01, **p < 0.05, *p < 0.1.

*Source*: see table 3.

^a^The *p*‐values refer to tests for significance of water capital, sewerage capital, the interaction of the two, and the joint significance of all of these on the basis of a wild bootstrap algorithm that clusters the standard errors at the town level.

^b^This is the percentage of the decline in the mortality rate that can be explained by the point estimate on the water and sewerage capital variables, respectively.

^c^This reports the Kleibergen–Paap rk Wald *F*‐statistics for the first stage of the LIML estimation reported in column (7). Stock–Yogo weak ID test critical values for 10% maximal IV size is 7.03.

^d^The selection ratio reports the delta value representing the degree of selection on unobservables relative to observables that would be necessary to explain away the result related to sewer capital as bias.

The specification in column (3) includes a comprehensive set of control variables and common time effects and estimates the effect of water and sewerage capital from within‐urban‐district variation over time. That is, it answers the question: how does a change in sewerage (water) capital over the past five‐year period *within* an urban district impact the change in all‐cause mortality in the current five‐year period *within* that district? The fact that we lag the sanitation variables by one five‐year period reduces the risk of reverse causality, but does not rule out omitted variables or attenuation bias. While the latter biases the point estimates towards zero, the negative relationship between sewerage capital and mortality could be driven by unobserved urban district‐specific time‐varying factors that happen to be negatively correlated with mortality and positively correlated with sewerage capital within a district. We adopt the method developed by Altonji et al. and extended by Oster to evaluate how concerning this is.^57^ Under the assumption that the observed factors included in the regression model are proportional to selection on the unobserved factors not in the model, we can evaluate the size of bias by calculating the selection ratio reported at the bottom of the table. In column (3), the selection ratio is 1.11. This means that the unobserved factors of concern need to be at least 1.11 times as important as the observed control variables and fixed effects to explain the results we find. To engage with omitted variables or attenuation bias, in column (7) we report a specification where we instrument (the lag of) water and sewerage capital with their lagged values. These instruments fail to pass the Kleibergen–Paap rk Wald test for weak instruments, and we estimate the model with limited maximum likelihood which is somewhat robust to that. We see that the effect of sewerage capital is a little bigger than in column (3) and can explain about 22 per cent of the decline in mortality over the period. The coefficient on the water capital variable is much (four) times larger than in column (3) and significant at the 10 per cent level. These results suggest that attenuation bias may be a problem and could be the reason why we find an insignificant result for any *direct* effect of water capital in most specifications estimated with ordinary least squares (OLS). On the other hand, the high selection ratio along with an IV estimate that is similar in magnitude to the OLS estimate lessen the concern that the estimated effect of sewerage capital on mortality is spurious.

**FIGURE 9:**
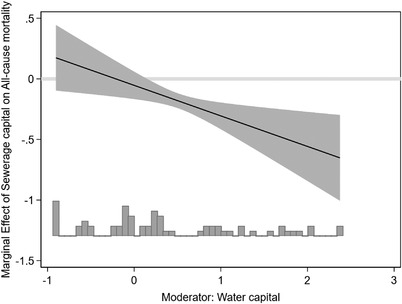
Complementarity between sewerage and water capital: the interaction effect illustrated. *Notes*: The underlying regression is reported in table 4, column 4. The grey bar indicates the distribution of observations; the solid line shows the marginal effect on all‐course mortality of additional sewerage capital conditional on water capital. The shaded area shows the 95 percent confidence interval around the point estimate. The capital stock variables are standardised, so they are deviations from the mean. Therefore water capital measured on the horizontal axis is sometimes negative: this occurs for observations below the mean. The effect of a one standard deviation increase in sewerage capital when water capital is at its mean value is located at 0 on the x‐axis and is −0.16. *Source*: See table 4

These results suggest that sewerage capital contributed to the mortality decline from 1880 onwards but that a direct contribution of water capital is absent or masked by attenuation bias. This, however, leaves an important question unanswered: did water capital reinforce the benign effect of sewerage capital and play an important *indirect* role, as suggested by the work by Alsan and Goldin?^58^ That is, is there evidence of complementarity between the two capital stocks? The specification in column (4) augments equation 2 with the interaction between water and sewerage capital to address this question.^59^ The coefficient on the interaction term is negative and significant at the 5 per cent level. Figure 9 plots the marginal effect of sewerage capital as a function of water capital, along with a histogram showing the distributions of the moderating variable.^60^ For low levels of water capital, additional sewerage capital has no effect on mortality, but once sufficient water capital is in place, the marginal effect becomes negative and significant. This is consistent with complementarity between the two capital stocks and suggests that water capital played an *indirect* role in generating the post‐1880 mortality decline even if we cannot find conclusive evidence of a direct or unconditional effect.

Columns (5) and (6) show the estimates for the balanced panel of 16 towns that includes urban district‐quinquennials with private ownership of waterworks and consequent underreporting in the public accounts of investment in water infrastructure. We see that the estimates are very similar to those reported in columns (3) and (4) for the 11 urban districts with municipalised water. In these specifications, we include a variable that measures the fraction of each five‐year period that an urban district had municipalised water supply. It has been argued that municipalisation correlates with the quality of water supply in this period.^61^ The estimates, however, suggest that municipalisation of water per se is unrelated to the decline in all‐cause mortality in the 16 largest urban districts between 1880 and 1909. Supplementary appendix tables A3 and A4 show that the results in table 4 are robust to excluding Salford, which had low public investment in water because it got its supply from nearby Manchester, to not weighting with population size, to outliers, to depreciating the capital stocks, and to a logarithmic transformation of the all‐cause mortality rate.

Table 5 reports the results of estimating equation 2 with diarrhoeal and infant mortality rates as the outcomes. These are often considered to be particularly related to sanitary conditions. We observe that the point estimates for sewerage capital are negative, but very noisily estimated and not significant except for the infant mortality rate in the specification without time fixed effects (column 4). In supplementary appendix table A5, we report further results for typhus and typhoid mortality rates, but recall from section III that these data measure mortality for different spatial units than the accounts data. We do not find significant negative effects of water capital for any of these mortality outcomes. These results confirm the findings reported in table 4, that investments in water provision had no measurable *direct* effect on mortality rates. In the case of typhoid (often regarded as a sensitive indicator of water quality), the point estimates for sewerage capital are negative, but not significant. This suggests that any effects of sewerage investments on crude mortality rates (table 4) operated via mechanisms other than or additional to effects on (measured) diarrhoeal or typhoid mortality.

**TABLE 5:** Regression results for diarrhoea and infant mortality rates (1880–1909), for the *Local Taxation Returns* sample

	Diarrhoea mortality rate			Infant mortality rate		
Dependent variable	(1)	(2)	(3)	(4)	(5)	(6)
Water capital (WC) t−1	−0.39 (−1.39)	0.011 (0.060)	−0.091 (−0.57)	−0.017 (−0.071)	0.030 (0.22)	−0.027 (−0.22)
Sewerage capital (SC) t−1	−0.28 (−1.16)	−0.068 (−0.28)	−0.10 (−0.46)	−0.66*** (−4.94)	−0.32 (−1.56)	−0.34 (−1.71)
WC × SC interaction t−1			0.20 (1.06)			0.11 (0.56)
Observations	63	63	63	63	63	63
*R* ^2^	0.567	0.877	0.885	0.783	0.914	0.916
Towns	11	11	11	11	11	11
Town FE	YES	YES	YES	YES	YES	YES
Time FE	NO	YES	YES	NO	YES	YES
Controls	YES	YES	YES	YES	YES	YES
Method	OLS	OLS	OLS	OLS	OLS	OLS
Period	1880–1909	1880–1909	1880–1909	1880–1909	1880–1909	1880–1909
Standard errors	Clustered	Clustered	Clustered	Clustered	Clustered	Clustered
*p*‐value (water)^a^	0.76	0.96	0.76	0.95	0.89	0.83
*p*‐value (sewerage)^a^	0.38	0.95	0.93	0.058	0.60	0.58
*p*‐value (joint)^a^	0.76	0.99	0.96	0.065	0.61	0.74
*p*‐value (inter)^a^			0.50			0.66

*Notes*: Diarrhoeal and infant mortality rates are per 1000 births. Control variables are tax base, population growth, female, aged 0–14, aged 15–44, birth rate, manufacturing employment, and textiles employment. All coefficients are standardised. Standard errors are clustered at the town level; *t*‐statistics given in parentheses. *R* ^2^ calculated for within variation. Regressions are weighted by average population over the period. All variables are measured at the urban district level. ***p < 0.01, **p < 0.05, *p < 0.1.

*Source*: see table 3.

^a^The *p*‐values refer to tests for significance of water capital, sewerage capital, the interaction of the two, and the joint significance of all of these on the basis of a wild bootstrap algorithm that clusters the standard errors at the town level.

Table 6 reports specifications of equation 2 with the stock of total outstanding debt (real per capita) as the proxy for sanitary investment. Since accounts data for debt are available for most of the 16 urban districts from 1867, this allows us to consider a longer time span and, since this is the explanatory variable used by Chapman, to compare our results with his. The downsides are discussed in section II.

**TABLE 6:** All‐cause mortality and debt per capita: regression results for different geographical units and periods of measurement

	All‐cause mortality rate per 1000 population						
Dependent variable	(1)	(2)	(3)	(4)	(5)	(6)	(7)
Debt square root	−0.091 (−1.59)	−0.087 (−1.68)	−0.10 (−1.17)	−0.33* (−2.13)	−0.30*** (−2.96)	−0.60* (−1.82)	−0.40 (−0.69)
Observations^a^	94	48	48	61	77	61	48
*R* ^2^	0.937	0.980	0.957	0.927	0.960	0.949	0.950
Towns	16	16	16	16	16	16	16
Town FE	YES	YES	YES	YES	YES	YES	YES
Time FE	YES	YES	YES	YES	YES	YES	YES
Controls	YES	YES	YES	YES	YES	YES	YES
Method	OLS	OLS	OLS	OLS	OLS	LIML	LIML
Period	1871–1900	1871–1900	1871–1900	1861–1900	1861–1910	1871–1910	1881–1910
Standard errors	Clustered	Clustered	Clustered	Clustered	Clustered	Robust	Robust
Unit	UD	UD	RD	RD	RD	RD	RD
Frequency	Five	Decennial	Decennial	Decennial	Decennial	Decennial	Decennial
*p*‐value (debt)^b^	0.12	0.16	0.27	0.059	0.0003	0.01	0.42
Decline explained^c^	11.6	9.52	11.2	35.3	26.6	52.7	34.6
K–P *F*‐statistic^d^						6.77	3.81
Selection ratio^e^				0.35	0.51		

*Notes*: Debt square root is the square root of real total outstanding debt per capita. UD indicates variables are measured over an urban district, RD indicates they are measured over a registration district. For the independent variables, data from the accounts (debt and tax base) are only available at the UD level, and these are, therefore, always for UDs. Data from the census and the mortality data are calculated either at the UD or RD level. Control variables are tax base, population growth, female, aged 0–14, aged 15–44, birth rate, manufacturing employment, and textiles employment. All coefficients are standardised. Standard errors are clustered at the town level or robust as indicated and the corresponding *t*‐statistics reported in parentheses. *R* ^2^ calculated for within variation. Columns (6) and (7) report results where we use the lagged value of the dependent variable as an instrument. The instruments are weak and we estimate with LIML. In column (6), we use the data points from the late 1860s to instrument debt in the 1870s; in column (7), we do not use the data from the 1860s. ***p < 0.01, **p < 0.05, *p < 0.1.

*Source*: See table 3.

^a^Debt data were unavailable for a number of towns in the early Local Taxation Returns, or pertained to different units from the later urban districts. In our sample, data for Birmingham, Manchester, and Newcastle Local Boards were unavailable for the 1860s and were omitted from our decennial panel for that decade.

^b^The *p*‐value refers to test for significance of debt square root on the basis of a wild bootstrap algorithm that clusters the standard errors at the town level.

^c^This is the percentage of the decline in the mortality rate that can be explained by the point estimate of debt square root.

^d^This reports the Kleibergen–Paap rk Wald *F*‐statistics for the first stage of the LIML estimation reported in columns (6) and (7). Stock–Yogo weak ID test critical value for 10% maximal IV size is 16.38.

^e^The selection ratio reports the delta value representing the degree of selection on unobservables relative to observables that would be necessary to explain away the result related to debt square root as bias. Reported only for significant variables.

We estimate equation 2 with data at different frequencies (five‐year periods or decennial) and geographical units (UD or RD). We estimate the effect of the square root of debt outstanding on all‐cause mortality to match Chapman's baseline specification.^62^ Column (1) is most similar to our baseline specification in table 4, column (3), with the only differences being: the independent variable is debt outstanding square‐rooted, the time period is 1871–1900 (reflecting the earlier availability of the total debt data, and the end date adjusted to match Chapman's), and the regressions are unweighted (as in Chapman's specifications). Importantly, the geographical units for the mortality rate and water and sewerage capital are the Urban Districts. Unlike our baseline regression, the result is insignificant. In columns (2) and (3), we report specifications with the mortality outcome and all controls (other than any from the *Local Taxation Returns*) measured at either the urban district (column 2) or at the RD level (column 3) to evaluate how important the issue of spatial units is. Both outcome and independent variables are decennial averages for the decades 1871–1900. In both specifications, the effect is insignificant and the decline explained is small. That is, using either our own geographical units or Chapman's, we could not replicate Chapman's results for our 16 towns for the period 1871–1900. Table 6, column (4) repeats the analysis in column (3), for all‐cause mortality in RDs at the decennial frequency, but for the *same* decades analysed by Chapman, that is, 1861–1900. With the decade 1861–70 included, debt has a significant negative effect on mortality and an explained decline of 35 per cent. This is comparable to Chapman's baseline result of 29 per cent of the decline explained. Column (5) shows a specification where we extend the sample to 1910 which produces a more precise estimate of the effects of total debt on mortality. Columns (6) and (7) report results from an instrumental variables (IVs) estimation where we instrument debt with its own lag. The instrument is weak, so we should be careful with the interpretation. The specification in column (6), which makes use of data from 1861 to 1910, exhibits a significant coefficient on debt that is twice the size of the corresponding OLS estimate in column (5). Column (7) leaves out the data from the 1860s, and we see that the IV estimate turns insignificant. To summarise, for our sample of the 16 largest urban districts, we *can* replicate Chapman's results when we include the decade of the 1860s in the analysis. Debt data, however, were only reported in the *Local Taxation Returns* from 1867 onwards, and so ‘average’ decennial outstanding debt was probably mis‐measured for the 1860s.^63^ Combined with the fact that we cannot find a *direct* effect of water capital post‐1880, this suggests that early investments in water, missed in our analysis, may have had disproportionately large effects, at least in our 16 towns, and that the role of water from the 1880s onwards was primarily to increase the value of investments in sewerage capital through complementarities. To investigate the question of early investments in water further, we turn our attention to the *Loans* sample.

Table 7 reports the results of estimating equation 2 with the infant mortality rate for the period 1845–84 as the outcome variable and the water capital variable constructed from Harris and Hinde's loans data for Manchester, Salford, Leeds, and Liverpool as the main explanatory variable. The first four columns build up the regression by progressively adding controls and fixed effects. We see that the raw correlation between water capital and infant mortality is negative (column 1) and that this is robust to adding town fixed effects (column 2), time fixed effects (column 3), and population growth, and the birth rate as control variables (column 4). In the full specification in column (4), the negative correlation between lagged water capital and infant mortality is significant, even with the wild bootstrapped standard errors, and the effect is substantial, being able to explain 39 per cent of the decline over the period 1845–84. The selection ratio suggests that omitted confounders need to be 1.40 times as important as the controls included for this effect to be due to bias. Column (5) reports an IV specification where (the lag of) water capital is instrumented by its own past value. This instrument is strong and the second stage estimate is a bit larger than the OLS estimate. Column (6) shows a specification where we have added mortality data pertaining to 1838–44 and in column (7), we have excluded the quinquennials with private waterworks. It makes little difference to the point estimate. In supplementary appendix table A6, we show that these results are robust to excluding Salford, to not weighting the data with population, to outliers, to depreciating the water capital stock, and to a logarithmic transformation of the infant mortality rate.

**TABLE 7:** Infant mortality (1845–84) and water capital: regression results from the *Loans* sample

	Infant mortality rate per 1000 births						
Dependent variable	(1)	(2)	(3)	(4)	(5)	(6)	(7)
Water capital, t‐1	−1.12*** (−4.43)	−1.71*** (−14.6)	−1.25*** −10.1)	−1.23*** (−8.93)	−1.38*** (−4.85)	−1.29*** (−14.3)	−1.19** (−3.34)
Observations	32	32	32	32	32	36	24
*R* ^2^	0.211	0.501	0.814	0.845	0.843	0.823	0.862
Town FE	NO	YES	YES	YES	YES	YES	YES
Time FE	NO	NO	YES	YES	YES	YES	YES
Controls	NO	NO	NO	YES	YES	YES	YES
Method	OLS	OLS	OLS	OLS	IV	OLS	YES
Period	1845–84	1845–84	1845–84	1845–84	1845–84	1840–84^a^	1855–84^b^
*p*‐value^c^	0.36	0.18	0.17	0.060	0.030	0.035	0.056
Decline explained^d^	35.4	54.0	39.5	38.8	43.6	40.6	37.6
Towns	4	4	4	4	4	4	4
K–P *F*‐statistic^e^					90.1		
Selection ratio^f^				1.40		1.21	1.70

*Notes*: Data for water capital pertain to 1840–84 but as we lag the variable, we model mortality over the period 1845–84. Control variables are population growth and birth rate. All coefficients are standardised. *t*‐Statistics based on robust standard errors are given in parentheses. *R* ^2^ calculated for within variation, except in column (1). Regressions are weighted by average population in the urban district over the period. Water capital and population growth are measured at the urban district (UD) level while the infant mortality rate and birth rate are measured at the registration district (RD) level. ***p < 0.01, **p < 0.05, *p < 0.1.

*Source*: See table 3.

^a^The Registrar‐General reported an average infant mortality rate for the period 1838–44 prior to reporting annually from 1845. This average is included for the five‐year period 1840–44.

^b^The four towns had private waterworks until between 1848 and 1852. The effect of this is included in the period fixed effect in the baseline specification. We have excluded water stock data for the two five‐year periods (1840–4 and 1845–9) with private waterworks in column (7).

^c^The *p*‐values refer to test for significance of water capital on the basis of a wild bootstrap algorithm that clusters the standard errors at the town level.

^d^This is the percentage of the decline in the infant mortality rate that can be explained by the point estimate of the water capital variable.

^e^This reports the Kleibergen–Paap rk Wald *F*‐statistics for the first stage of the 2SLS estimation reported in column (5). Stock–Yogo weak ID test critical values: 10% maximal IV size 16.38. The instrument is the second lag of water capital.

^f^The selection ratio reports the delta value representing the degree of selection on unobservables relative to observables that would be necessary to explain away the result related to water capital as bias.

Table 8, columns (1) to (4) show a sequence of regressions where, starting from the period 1845–74, we progressively add two or three five‐year periods. In each column, we report three coefficients for water capital from three different regressions estimated on the same sub‐sample. The first row shows the results for the baseline with water capital lagged one five‐year period and estimated with OLS. In the second row, we lag water capital two periods to allow for a longer time lag (10–15 years) between when a loan was sanctioned to when it had an effect on mortality. The third row shows the IVs limited information maximum likelihood (LIML) estimate where the first lag of water capital is instrumented with the second lag. We observe a negative correlation between water capital and infant mortality for all subsamples irrespective of estimator and lag structure, but the point estimates become progressively three to six times smaller (numerically) as we add quinquennials to the sample. The estimates are generally imprecise, but statistically significant in the samples ending in 1874 and 1884 (columns 1 and 2) with robust standard errors, and in four out of six cases also with the wild bootstrapped clustered standard errors. The estimates are insignificant in the OLS specifications for the samples that end in 1894 and 1909 (columns 3 and 4), but remain significant (with robust, not with clustered standard errors) when the IVs estimator is employed. Column (5) shows a specification estimated on quinquennial data from 1875 to 1909. We see that water capital has a tiny and insignificant effect on mortality during that period. This suggests that the benign effect of investment in water supply operated up through the 1870s to the mid‐1880s, and if the effect continues beyond that, its magnitude was three to six times smaller than in the earlier period. This suggestive finding is consistent with the result that water capital did not exhibit a *direct* effect on mortality from 1880 onwards in the *Local Taxation Returns* sample (where we found the effect to be operating through complementarity with sewerage capital).

**TABLE 8:** Infant mortality and water capital: regression results for different time periods from the *Loans* sample

	Infant mortality rate per 1000 births				
Dependent variable	(1)	(2)	(3)	(4)	(5)
Water capital, t−1 (OLS)	−1.31* (−2.37)	−1.23*** (−8.93)	−0.40 (−1.10)	−0.45 (−1.76)	−0.099 (−0.75)
Water capital, t−2 (OLS)	−2.04*** (−6.86)	−1.94*** (−8.32)	−0.47 (−1.97)	−0.34 (−1.83)	0.022 (0.12)
Water capital, t−1 (LIML)	−2.19* (−2.17)	−1.38*** (−4.85)	−0.53* (−1.73)	−0.44** (−2.18)	0.045 (0.17)
Observations	24	32	40	52	28
*R* ^2^ (OLS, t−1)	0.661	0.845	0.759	0.824	0.790
*R* ^2^ (OLS, t−2)	0.700	0.857	0.767	0.804	0.785
Towns	4	4	4	4	4
Town FE	YES	YES	YES	YES	YES
Time FE	YES	YES	YES	YES	YES
Controls	YES	YES	YES	YES	YES
Period	1845–74	1845–84	1845–94	1845–1909	1875–1909
*p*‐value^a^ (OLS, t−1)	0.23	0.06	0.56	0.45	0.40
*p*‐value^a^ (OLS, t−2)	0.054	0.052	0.41	0.37	0.86
*p*‐value^a^ (LIML)	0.14	0.03	0.43	0.38	0.88
K–P F‐statistic^b^	25.0	90.1	60.0	94.8	10.8
Selection ratio^c^ (OLS, t−1)	0.66	1.40			
Selection ratio^c^ (OLS, t−2)	0.50	0.99			

*Notes*: The coefficients and *t*‐statistics reported in row (1) are estimated with OLS using the first lag of water capital; those in reported in row (2) are also estimated with OLS but using the second lag of water capital, and those reported in row (3) are estimated with limited information maximum likelihood using the second lag of water capital as the instrument. Control variables are population growth and birth rate. All coefficients are standardised. *t*‐Statistics based on robust standard errors are given in parentheses. *R* ^2^ calculated for within variation and reported for the OLS estimations. Water capital and population growth are measured at the urban district (UD) level while the infant mortality rate and birth rate are measured at the registration district (RD) level. ***p < 0.01, **p < 0.05, *p < 0.1.

*Source*: See table 3.

^a^The *p*‐values refer to test for significance of water capital on the basis of a wild bootstrap algorithm that clusters the standard errors at the town level.

^b^This reports the Kleibergen–Paap rk Wald *F*‐statistics for the first stage of the LIML estimations. Stock–Yogo weak ID test critical values: 10% maximal IV size 16.38. The instrument is the second lag of water capital and is strong in all specifications.

^c^The selection ratio reports the delta value representing the degree of selection on unobservables relative to observables that would be necessary to explain away the OLS result related to water capital as bias. Reported for the significant coefficients only.

Table 9 reports the results for four other public health outcomes for which we have constructed data from 1845 to 1884: early childhood mortality rate, crude death rate, life expectancy at birth, and life expectancy at age 15. For each outcome, we report specifications with two‐way fixed effects and controls estimated either with OLS or with an IVs estimator (LIML) using past values of water capital as the instrument. We observe that lagged water capital is negatively associated with early childhood mortality and the crude death rate and positively related to life expectancy at birth. It is not associated with life expectancy at age 15.

**TABLE 9:** Alternative public health indicators (1845–84) and water capital: regression results from the *Loans* sample

	Early childhood mortality rate		Crude death rate		Life expectancy at birth		Life expectancy at age 15	
Dependent variable	(1)	(2)	(3)	(4)	(5)	(6)	(7)	(8)
Water capital t−1	−0.51** (−3.64)	−0.47** (−2.46)	−0.41* (−2.43)	−0.44*** (−3.09)	0.58** (3.76)	0.64*** (3.42)	0.11 (0.40)	0.17 (0.75)
Observations	32	32	32	32	32	32	32	32
*R* ^2^	0.926	0.925	0.921	0.921	0.912	0.912	0.831	0.830
Towns	4	4	4	4	4	4	4	4
Towns FE	YES	YES	YES	YES	YES	YES	YES	YES
Time FE	YES	YES	YES	YES	YES	YES	YES	YES
Controls	YES	YES	YES	YES	YES	YES	YES	YES
Method	OLS	LIML	OLS	LIML	OLS	LIML	OLS	LIML
Period	1845–84	1845–84	1845–84	1845–84	1845–84	1845–84	1845–84	1845–84
*p*‐value^a^	0.17	0.095	0.27	0.35	0.19	0.092	0.72	0.83
Δ explained^b^	16.1	14.7	13.0	14.0	18.3	20.0	3.47	5.48
CD *F*‐statistic^c^		90.1		90.1		90.1		90.1
Selection ratio^d^	1.30		1.78		1.24			

*Notes*: Crude death rate is total deaths per 1000 population; early childhood mortality rate is the probability of dying between exact ages one and five years, per 1000 children surviving to exact age one. Control variables are population growth and crude birth rate. All coefficients are standardised. *t*‐Statistics based on robust standard errors are given in parentheses. *R* ^2^ calculated for within variation. Regressions are weighted by average population over the period. Water capital and population growth are measured at the urban district (UD) level while the mortality rates and life expectancy variables and birth rate are measured at the registration district (RD) level. ***p < 0.01, **p < 0.05, *p < 0.1.

*Source*: See table 3.

^a^The *p*‐values refer to test for significance of water capital based on a Wild bootstrap algorithm that clusters the standard errors at the town level.

^b^This is the percentage of the change (decline or increase) in the mortality rate or in life expectancy that can be explained by the (significant) point estimate of water capital variable in each regression, respectively.

^c^This reports the Craig–Donald *F*‐statistics for the first stage of the 2SLS estimation reported in columns (2), (4), (6), and (8). The instrument is the second lag of water capital.

^d^The selection ratio reports the delta value representing the degree of selection on unobservables relative to observables that would be necessary to explain away the result related to water capital as bias. Reported only for significant variables.

Taken together the results from tables 7, 8, 9 suggest that the stocks of water capital (funded via centrally sanctioned loans) helped reduce infant and childhood mortality with little effect on adult mortality, and, most importantly, that this effect was primarily concentrated in the period from the first Public Health Acts in the later 1840s to the mid‐1880s. This interpretation should, of course, be treated with caution: our sample is small and the spatial units for which public health outcomes and water capital are measured are mismatched. Nonetheless, the results highlight the importance of studying the early period in future research and challenge the view that all the action took place from 1870 onwards.

## V DISCUSSION

Debates regarding the importance of public investment in urban public health for mortality declines in British cities have been hampered by inadequate data. One particular problem has been the geographical mismatch between mortality data (available as decadal averages for RDs) and public investment data (available annually for urban districts). Another problem is that until recently it has been difficult to analyse the relationship between sanitary investments and the mortality decline for the critical period between the Public Health Act of 1848 and the 1870s. In this paper, we overcame the first problem by using mortality data for the 16 largest (non‐metropolitan) urban districts, and we engage with the second problem by drawing on data collected by Harris and Hinde on loans for public investment in water supply for the earlier period. In addition, we used refined measures of sanitation capital constructed using the perpetual inventory method from recorded capital expenditures in water and sewerage, in preference to data on aggregate debt. While our solution to the problem of geographical mismatch limited us to the study of only 16 towns, and we only have data on investment in water supply before 1875 for four towns, these were the largest towns and constituted a major component of the ‘urban penalty’ nationally in this period.

Our analysis sheds new light on the debate about municipal investment in public health and the mortality decline in urban England from 1845 to 1909, and although some of our conclusions must be viewed as tentative given the limited data at our disposal, we highlight the following insights. First, for the period 1880–1909 (where we were able to resolve the spatial mismatch problem and could distinguish between water and sewerage capital) we found that the stock of sewerage capital contributed significantly to improving health in the largest urban districts (outside London) and could explain about 13 per cent of the decline in all‐cause mortality. We could not, however, find evidence that sewerage investments affected (measured) diarrhoeal or typhoid mortality, which suggests that the benign effect on all‐cause mortality rates operated via other mechanisms. For water capital, on the other hand, we could only robustly detect an indirect effect, in that we found that the presence of water capital reinforced the capacity of sewerage capital to reduce mortality, suggesting complementarity between the two assets, while the direct effect was imprecisely estimated and insignificant in most specifications. This is in line with the evidence from Boston from 1880–1920 where Alsan and Goldin found that clean water and effective sewerage systems reinforced each other, accounting for one‐third of the under‐five mortality decline.^64^ Second, for the sample of four urban districts, we could study the effect of water‐related investments on mortality from 1845 onwards. We found a substantial reduction in infant and childhood mortality but no effect on adult mortality. Most importantly, this effect was concentrated in the period from the first Public Health Acts in the late 1840s to the mid‐1880s and our estimates suggest that the direct effect of water investment was much smaller after 1885.

Our findings relate to a small number of large urban districts; however, they are consistent with the timing of mortality declines at the national level (figure 2). The diseases most dependent on water for transmission (cholera and dysentery) had already declined substantially by the mid‐1880s. Recent work by Hinde and Harris has investigated the geographical patterning of these changes using cause‐specific decadal crude mortality rates in RDs. They report that typhoid and typhus rates declined by two‐thirds between the 1860s and the 1880s, and these declines were most rapid in the largest towns.^65^ Diarrhoeal diseases, that depend to a greater extent on hygienic handling of food and possibly on fly‐borne transmission, fell more modestly in the same decades, before stalling in the 1890s, and then falling again after 1900. Our results suggest an explanation for these patterns: the early efforts to supply clean drinking water by the filtration of river water, the sourcing of remote (unfiltered) upland water, and the provision of public standpipes were sufficient to cause substantial reductions in the transmission of water‐borne diseases. Indeed, these early efforts may have had disproportionately large effects on mortality because those diseases that depended most on contaminated water for transmission were also the most lethal (table 1). While further large investments were required to sustain the provision of water as urban populations grew (most notably in Birmingham, where the corporation was only forced to seek remote sources of water in the early twentieth century), these did not necessarily result in improvements in quality; rather, they were essential to prevent deterioration. Later refinements (such as the extension of filtration to all water sources and of piped supplies to all households) may then have faced diminishing returns, because the population already had access to relatively clean drinking water that reduced exposure to the most lethal diseases. Further increases in abundance and access to water may have improved health primarily through reinforcing the effectiveness of sewerage systems. Sewerage served to remove faeces from households and from urban environments, and therefore helped to reduce diseases that were transmitted primarily via unwashed hands and insects. However, the widespread adoption of flush toilets generally occurred much later and more gradually than the supply of clean water, and this may explain the late persistence of high rates of diarrhoea in many towns even as the more water‐borne diseases declined. The most sanitary type of toilet also required an abundant and in‐house supply of water, and this dependence may explain the synergy we observed between investments in sewerage and water. We could not, however, identify how sewerage reduced mortality in our study, given the limited available range of mortality variables.

Our analysis focused on the largest provincial towns, and these were some of the earliest to invest in water filtration and long‐distance supplies. If our hypothesis regarding the disproportionate effects of precocious investments in water is correct, then we would expect larger samples, such as Chapman's and that of Hinde and Harris, to reflect the heterogeneous and overlapping effects of early and late investments in large and small towns, and this may explain some of the discrepancies between their studies, and ours.^66^ The apparently early effects of clean water and late effects of sewerage on mortality reported here also call for greater attention in future studies to the sequencing of sanitary interventions, to their interactions, and to the multiplicity of potential pathways for disease transmission.

## Supporting information

Supporting Information

Appendixes tables A1–A6

Supporting Information

## Data Availability

Toke S. Aidt, Romola J. Davenport, and Felix Grey. New perspectives on the contribution of sanitary investments to mortality decline in English cities, 1845–1909: Replication data. Ann Arbor, MI: Inter‐university Consortium for Political and Social Research [distributor], 2022‐07‐12. https://doi.org/10.3886/E175041V1.

## Footnotes

1
McKeown and Record, ‘Reasons’.

2
Chapman, ‘Contribution of infrastructure’; idem, ‘Interest rates’.

3
Harris and Hinde, ‘Sanitary investment’; Hinde and Harris, ‘Mortality decline’.

4
Anderson et al., ‘Re‐examining’; Beach et al., ‘Typhoid fever’; Condran, ‘Declining mortality’; Cutler and Miller, ‘Role of public health improvements’, p. 13; Ferrie and Troesken, ‘Water’; Phillips et al., ‘Changes’.

5
Anderson et al., ‘Re‐examining’; Anderson et al., ‘Summer diarrhea’; Condran, ‘Declining mortality’.

6
Brown and Guanine, ‘Infant mortality decline’; Floris and Staub, ‘Swiss towns’; Helgertz and Önnerfors, ‘Public water’; Peltola and Saaritsa, ‘Later, smaller, better?’; van Poppel and van der Heijden, ‘Effects of water supply’.

7
de Looper et al., ‘Sanitary improvement’; Gallardo‐Albarrán, ‘Sanitary infrastructures’; Kesztenbaum and Rosenthal, ‘Sewers’ diffusion’; Peltola and Saaritsa, ‘Later, smaller, better?’.

8
de Looper et al., ‘Sanitary improvement’; Floris and Staub, ‘Swiss towns’.

9
Alsan and Goldin, ‘Watersheds’; Gallardo‐Albarrán, ‘Sanitary infrastructures’.

10
Hassan, ‘Growth and impact’.

11 Ibid.; Hassan, ‘Impact and development’.

12
Harris and Hinde, ‘Sanitary investment’, p. 9; Webster, ‘Public Works Loans Board’, pp. 128–59.

13
Hassan, ‘Growth and impact’; Szreter, ‘Importance of social intervention’, pp. 22–8; Szreter and Woolcock, ‘Health by association?’; Wohl, *Endangered lives*, pp. 181–2.

14
Chapman, ‘Contribution of infrastructure’, p. 235.

15 Ibid.

16
Chapman, ‘Interest rates’.

17 In contrast, Chapman, in ‘Contribution of infrastructure’, constructed only crude all‐cause or cause‐specific death rates, that is, deaths divided by the total population without any adjustment for age structure.

18
Hinde and Harris, ‘Mortality decline’.

19 See also Bell and Milward, ‘Public health expenditures’.

20
van Poppel and van der Heijden, ‘Effects of water supply’.

21
Ewald, ‘Waterborne transmission’; idem, ‘Evolution of virulence’.

22
Hardy, ‘Cholera’, pp. 159, 161–5; eisdem, *Salmonella*; Luckin, *Pollution and control*, pp. 134–5; Seaton, ‘Discussion’.

23
Davenport et al., ‘Cholera’, fig. 10.

24 On the anomalously high rates of typhoid in US cities in the early twentieth century, see Hardy, ‘Scientific strategy’. On the cumulative effects of the gradual extension of constant piped water in London, see Troesken et al., ‘Health benefits’.

25
Chapman, ‘Contribution of infrastructure’, pp. 237–40.

26 Feenstra et al,, ‘Next generation’.

27
*Local Taxation Returns* (P.P. 1867–1913). Throughout this paper we use the year in which a town's fiscal year ends to refer to the entire fiscal year.

28 The 16 cities are: Birmingham, Bradford, Bristol, Hull, Leeds, Leicester, Liverpool, Manchester, Newcastle, Norwich, Nottingham, Portsmouth, Salford, Sheffield, Sunderland, and Wolverhampton.

29 London had a different structure of local government from the rest of England in this period and the close proximity of the vestries and the significant network externalities associated with both water supply and sewerage systems make it complicated to study the link between public investment in sanitation and public health outcomes in London. We do not attempt to do so in this paper.

30 These were Local Boards or Improvement Commissioners up until 1872, Urban Sanitary Districts in the period 1873–1895, and Urban Districts from 1896. We use the term urban district (UD) to describe these urban units.

31 This choice affects the level of the resulting capital stocks which may be mis‐measured if, for example, repayment schedules were town‐specific. For the purpose of our statistical analysis, however, this is not a problem, as the town fixed effects adjust for this, but it is a potential problem for between‐town comparisons of levels.

32 Supplementary appendix table A4.

33
Harris and Hinde, ‘Sanitary investment’. We are very grateful to these authors for their generosity in sharing these data.

34 To ensure comparability with the *Local Taxation Returns* sample, we do not depreciate the stock in the baseline, but see supplementary appendix table A6 for results with a positive depreciation rate.

35 The towns are Liverpool, Leeds, Manchester (with Prestwich), and Salford.

36 For example, in Liverpool a loan of £400 000 was sanctioned in 1847 for the municipal acquisition of the waterworks (acquired 1848); for the extension of pipes and consolidation of water supplies (which proceeded from 1851); and for the sourcing of water from Rivington Pike, which first became available in 1857. In 1880 a further loan of £3 250 000 was approved for construction of the Vyrnwy reservoir in north Wales, and the first water was delivered from the reservoir in 1892; Bradford, ‘Historical approaches to uncertainty’, p. 24; Sheard, ‘Water and health’.

37
Hassan and Taylor, ‘Politics of water’; Silverthorne, *Purchase of gas and waterworks*, p. 70, but see also Beach et al., ‘Who should own?’.

38 The estimates of the capital stocks from the *Local Taxation Returns* are based on the capital expenditure data while the water stock estimate from the *Loans* sample is based on loans sanctioned. The level of the water capital stock constructed from the two sources differs (figure 4), and this reflects (1) the full or partial repayment of loans taken out before 1875; (2) private loans sought or approved before municipalisation; (3) delays in the implementation of sanctioned loans (we have no evidence to suggest that loans were sanctioned but not used at all); and (4) the perpetual inventory method requiring an anchoring point, which is different in the two cases. The levels of the two series are, therefore, not directly comparable and the focus should be on the trends. Note that values for Salford are very low in both sources.

39 Local Government Board, *Water Undertakings* (P.P. 1914, LXXIX), p. xxvii.

40
Sheail, ‘Town wastes’.

41
Hardy, *Salmonella*, chap. 2; Royal Commission on Sewage Disposal, *Interim Report* and idem, *Fifth Report*.

42 The Registrar‐General also reported a more limited set of mortality statistics for registration sub‐districts (table 2). These were sub‐divisions of RDs (comprising over 2000 in total, compared with approximately 630 RDs), but were never designed to distinguish urban from rural districts.

43 See footnote 29 for why we exclude London.

44 In cases where a boundary change occurred between census dates, we used the ratio of the number of (weekly) births in the 52 weeks before the boundary change to the number in the 52 weeks following the change to estimate the change in population. We then used the intercensal growth rate in the decade closest to the change to either forward‐project or back‐project the nearest census population to the date of the boundary change to estimate the population at the date of change. We estimated the population before or after that date from the ratio of births before and after the boundary change.

45 Deaths for the years 1838–44 were reported in aggregate, not annually. We do not include these data in the main analysis, but report results with them in table 7.

46 For 1841 populations by age and sex in RDs were reported in the *9th Annual Report of the Registrar‐General*.

47
Hanlon et al., ‘Temperature, disease and death’; Woods et al., ‘Causes’.

48 This discrepancy may reflect the impact of adult migration into especially Liverpool and Manchester during the ‘cotton famine’, which is captured in our counts of annual deaths but not in the population denominators of the death rates (which are derived from interpolation between census dates). Infant mortality rates are calculated using reported annual births and deaths and so are less liable to distortions caused by intercensal migration.

49 The mortality data cover the period 1880–1909, and the (lagged) capital data the period 1875–1905.

50
Chapman, ‘Contribution of infrastructure’.

51
Bertrand et al., ‘How much?’

52
Arellano, ‘Computing’.

53
Cameron and Miller, ‘Practitioner's guide’.

54
Roodman et al., ‘Fast and wild’.

55 Supplementary appendix table A2 reports the coefficients on the control variables.

56 We calculate the ‘decline explained’ as: $Decline\;explained=\;\frac{\frac{1}{T}\sum_{t}^{T}\sum_{i}P_{i,t}{\hat{\beta }}_{j}\left(S_{i,t}-S_{i,t-1}\right)}{\frac{1}{T}\sum_{t}^{T}\sum_{i}P_{i,t}\left(M_{i,t}-M_{i,t-1}\right)}$, where *P_i,t_* is population in urban district *i* in period *t*, ${\hat{\beta }}_{j}\;$ is the estimated regression coefficient on the relevant sanitation variable, $S_{i,t}^{j}$, is the associated measure of either water or sewerage capital, and *M_i,t_* is the mortality rate in period *t*. This is in spirit of the formula Chapman, ‘Contribution of infrastructure’ reports (equation 2, p. 244), but different in that he uses final infrastructure over the change in mortality, rather than the change in infrastructure over the change in mortality. In addition, we divide the period into sub‐intervals and take the average of the weighted change over these intervals. This gives a more realistic estimate of the decline explained than simply doing a linear projection over all years.

57
Altonji et al., ‘Selection’; Oster, ‘Unobservable selection’.

58
Alsan and Goldin, ‘Watersheds’.

59 Supplementary appendix table A2, columns (2) to (5) report regressions that progressively add controls and fixed effects. The size of the interaction term is stable and significant in all specifications.

60
Hainmueller et al., ‘How much?’. Water and sewerage capital (along with all the other variables) are standardised to have zero mean. Thus, negative (positive) values of the two capital stocks refer to values below (above) the mean. The appropriate interpretation of the coefficients on the sewerage and water capital variables (0.055 and −0.16, respectively) is, therefore, the marginal effect of the variable when the other variable is at its mean.

61
Hassan, ‘Growth and impact’.

62 We take table 1, column (3) to be Chapman's baseline result; Chapman, ‘Contribution of infrastructure’, p. 244. He finds the square root of loans outstanding has a standardised coefficient of −0.17, significant at 1%. The ‘% decline explained’ by loans is 29%.

63
*Loans* data were unavailable for a number of towns in the early *Local Taxation Returns*, or pertained to different units from the later urban districts. In our sample, data for Birmingham, Manchester, and Newcastle Local Boards were unavailable for the 1860s and were omitted from our decennial panel for that decade.

64
Alsan and Goldin, ‘Watersheds’.

65
Hinde and Harris, ‘Mortality decline’.

66 Recent work by Chapman indicates that high interest rates before the mid‐1880s exerted a significant brake on municipal borrowing, and that this effect was most marked in smaller towns; Chapman, ‘Interest rates’.
