# Supplementary material for: New perspectives on the contribution of sanitary investments to mortality decline in English cities, 1845–1909
Source: Econ Hist Rev. 2022 Sep 26;76(2):624–60. doi: 10.1111/ehr.13195 (PMC10952366; doi:10.1111/ehr.13195)
Supplement: Supplementary file 1 — Supporting Information [file EHR-76-624-s001.docx]

**Appendix A - Data construction**

# Construction of the water and sewerage capital stocks

As explained in Section II of the main text, we use the Perpetual Inventory Method to estimate the stocks of water and sewerage capital in each town on a yearly frequency (which we then aggregate to five-year periods for the purpose of the statistical analysis) for the *Local Taxation Returns* sample. To do this we need three inputs: a time series of capital expenditures, the initial capital stock and the depreciation rate. This appendix explains how we get these inputs from the data available.

*Capital expenditure*

For the period 1883 onwards, we use expenditures defrayed from loans which are recorded in the accounts to measure the investment expenditure in that year. In order to obtain a series of capital expenditure data going back to 1875 (or 1872), it was necessary to estimate capital expenditure because only combined (current and capital) expenditure was reported in the *Local Taxation Returns*. To see the logic behind our approach, suppose that that current spending on maintenance and running is approximately proportional to the stock of capital, i.e., the bigger the stock the more it cost to keep it running. The stock at time t is the sum of past investment or capital expenditures $\sum_{j=0}^{t} I_{ij}$=$\sum_{j=0}^{t-1} I_{ij}+I_{it}$ where $I_{it}$ is investment a given capital stock in town *i* in year *t* and we split out the current addition to the stock from those of the past. Given this, we can write

$\frac{C_{it}}{\sum_{j=0}^{t-1} I_{ij}+I_{it}}=K_{i}$ (1)

where $K_{i}$ is the town-specific factor of proportionality (a small positive number) and $C_{it}$ is current expenditure. We can then write the relationship between total spending $T_{it}$ and investment $I_{it}$ at time t as

$C_{it}+I_{it}=T_{it}.$ (2)

We can rewrite this by substitution of equation (1) into equation (2) as

$I_{it}=-\frac{K_{i}}{1+K_{i}}\sum_{j=0}^{t-1} I_{ij}+\frac{1}{1+K_{i}}T_{it}.$ (3)

Equation (3) gives a simple town-specific relationship between total expenditure in a given year and the associated capital expenditure. If the proportionality assumption does not hold exactly, then the relationship will not be linear as in equation (3) and to account for that we estimate regressions of the following type

$I_{it}=F_{i}(T_{it})+\omega_{it}$ (5)

where the function *F_i_* is specific to town *i* and $\omega_{it}$ is an error term. We estimated this relationship between capital expenditure *I_it_* and combined (current and capital) expenditure *T_it_* during a period (defined below) for which it was disaggregated. We tested a large number of possible functions *F_i_*: linear, quadratic or cubic in *T_it_*; including controls for the change in town *i*'s tax base or total loans outstanding; and including up to five years of leads in *T_it_* (since future current spending may be a good predictor of today’s capital spending); and all possible combinations of these. We then calculated the predicted values for capital investment using the estimated function *F_i_*. We assessed out-of-sample fit by calculating the residual sum of squares (RSS):

|  | $RSS=\sum_{i} \sum_{t} (I_{it}-\hat{I}_{it})^{2}$. | (6) |
| --- | --- | --- |

We tested two possible sets of time periods for the estimation and out-of-sample evaluation. First, motivated by the desire to capture the relationship between capital and total spending as temporally close to the period 1875-82 as possible, we chose the 10-year window 1883-1992 to estimate the model and then considered the out-of-sample performance of the model for the ten years after that, 1893-1902. Second, motivated by the desire to use the whole sample period, we estimated the model over all but the last ten years of available data, 1883-1902, and then assessed out-of-sample performance for the final ten years, 1904-1913.

The results were as follows and summarized in Table S1. When estimating over 1883-1892, for water and sewerage, the function *F_i_* that gave the lowest out-of-sample RSS for both water and sewerage spending included a linear, quadratic and cubic in *T_it_*, two leads in *T_it_*, and no other controls. When estimating over 1883-1902, this remained true, but the RSS (per out-of-sample year) was considerably higher. We therefore chose to use the above function *F_i_* estimated over 1883-1892 when calculating the estimated capital expenditure for the period 1875-1882 to be used in our analysis (not shown in the Table). Figures S1 (sewerage investments) and S2 (water investment) show time plots of the predicted values (the solid lines) for the estimation period till 1892 and for seven years out of sample till 1900 for sewerage capital expenditures and for the seven Urban Districts that had municipalized water from 1875; otherwise for the number of years with municipalized water between 1872 and 1883. The plots also graph the actual capital expenditure values for the same period (the dashed lines). All data are in real per capita units. We observe that the estimated model has a very good within sample fit (1883-1892). For some of the towns the out of sample fit is also remarkably good, e.g., Liverpool. For others, the fit is less good, as in the case of Birmingham’s water investment in the 1890s which the link between total expenditure on water and capital expenditures for the 1880s do not predict.

On the assumption that the estimated relationship holds also over the period 1875-1882, we then estimate capital expenditures for water and capital separately for these years according to:

|  | $\hat{I}_{it}=\max_{} \left\{ \hat{F}_{i}(T_{it}),0 \right\},$ | (7) |
| --- | --- | --- |

where the estimated *F* function is specific to the underlying capital asset (water or sewerage) and town. That is, the imputed investment in district *i* in year *t* is the predicted value from equation (5) if it is positive and zero otherwise. The number of “zeros” for the 8 years that are constructed is 66 and 54, respectively for water and sewerage capital. The corresponding numbers for the first 8 years (1883-90) with actual capital expenditure data are 55 and 42. This does not suggest that the imputed series are problematic in this regard. This gives us a panel of capital expenditures for water (1875-1910) and for (1872-1910) for sewerage from which we can construct the underlying capital stocks.

Table S1: Selected specifications of the $F_{i}\left( T_{it} \right)$ function and the out of sample RSS calculations.

| Model | No. of leads | Linear | Square | Cube | Controls | Out of sample RSS  Water investment | Out of sample RSS  Sewerage investment |
| --- | --- | --- | --- | --- | --- | --- | --- |
| 1 | 0 | YES | NO | NO | NO | 3.60 | 3.47 |
| 2 | 0 | YES | YES | NO | NO | 4.19 | 2.74 |
| 3 | 0 | YES | YES | YES | NO | 3.13 | 2.38 |
| 4 | 0 | YES | NO | NO | GROUP 1 | 2.59 | 2.43 |
| 5 | 0 | YES | NO | NO | GROUP 2 | 3.25 | 2.93 |
| 6 | 0 | YES | NO | NO | GROUP 3 | 2.68 | 3.66 |
| 7 | 0 | YES | NO | NO | GROUP 1-3 | 1.82 | 2.52 |
| 8 | 2 | YES | NO | NO | NO | 3.26 | 2.27 |
| 9 | 2 | YES | YES | NO | NO | 1.97 | 2.03 |
| 10 | 2 | YES | YES | YES | NO | **1.46** | **1.57** |
| 11 | 3 | YES | YES | YES | NO | 1.83 | 1.58 |
| 12 | 4 | YES | YES | YES | NO | 18.69 | 1.88 |
| 13 | 5 | YES | YES | YES | NO | 52.0 | 2.44 |

Note: The out of sample RSS calculation is explained in the text. Control group 1 is total real receipts per capita; control group 2 is the change in real outstanding debt per capita; and control group 3 is change in real rateable values per capita.

Figure S1: Within and out of sample predicted sewerage-related capital expenditures per capita and actual real capital expenditures per capita, 1883-1900.


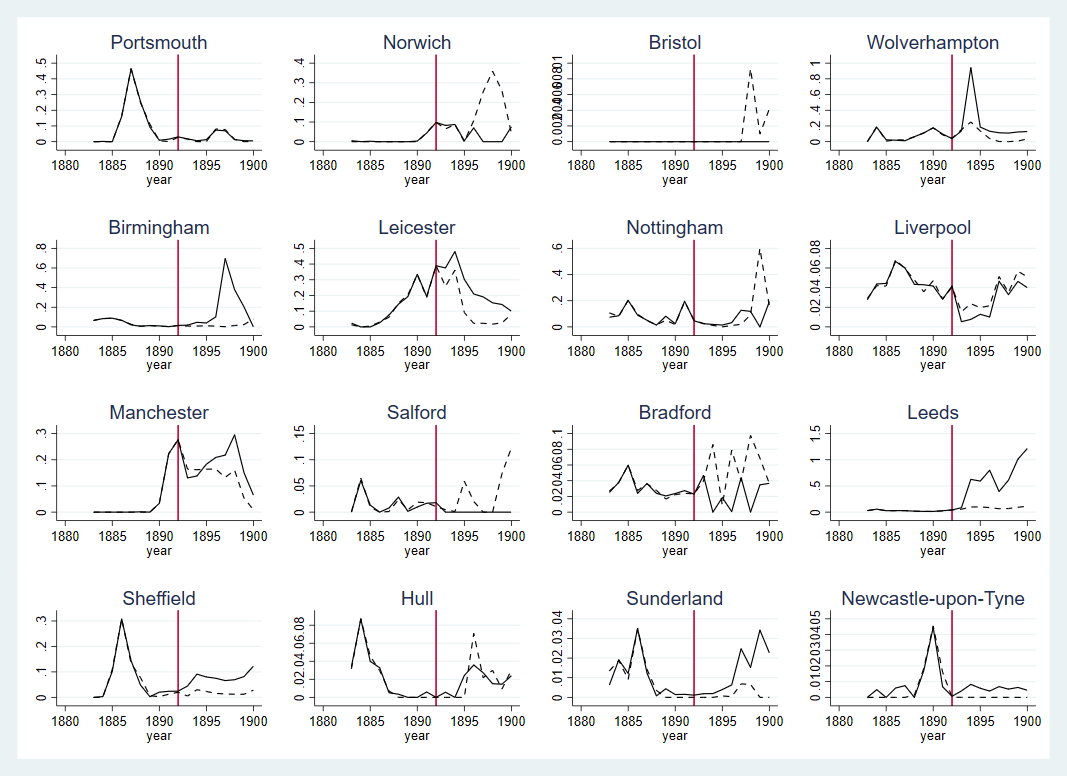


*Notes*: The solid line is the predicted value for sewerage-related capital expenditures per capita; the dotted line is the actual values. The vertical line demarcates 1892; observations to the left are within the estimation window while observations to the right are out of sample.

Figure S2: Within and out of sample predicted water-related capital expenditures per capita and actual real capital expenditures per capita, 1883-1900.


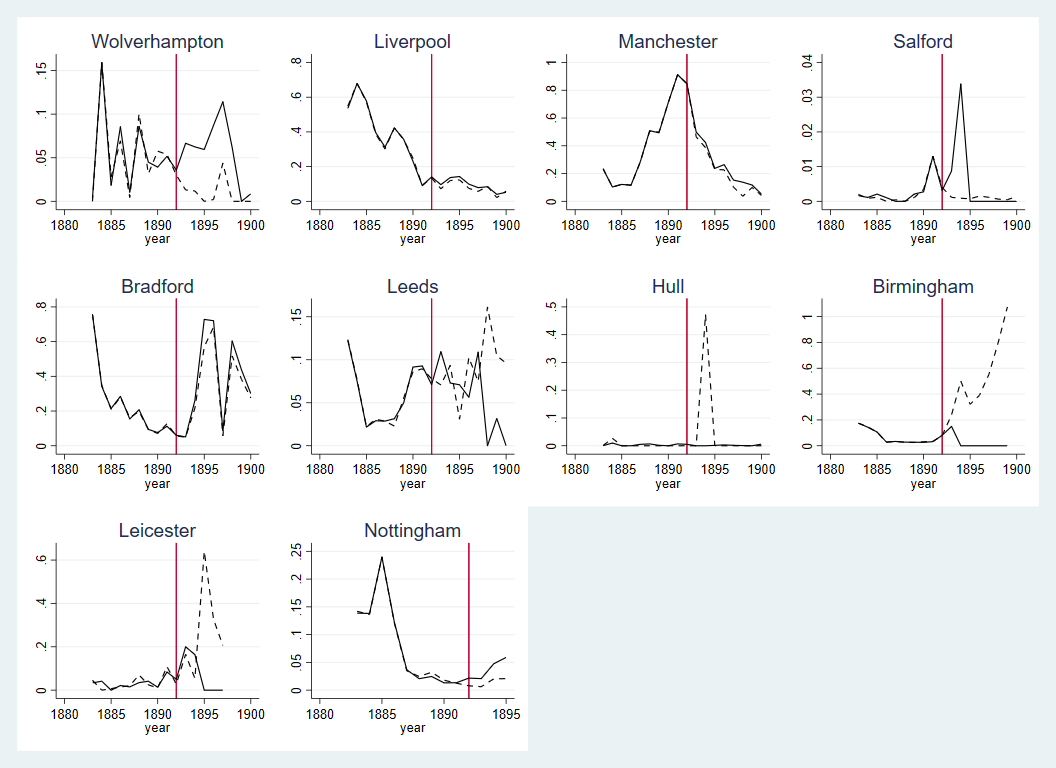


*Notes*: The solid line is the predicted value for sewerage-related capital expenditures per capita; the dotted line is the actual values. The vertical line demarcates 1892; observations to the left are within the estimation window while observations to the right are out of sample. We only graph the data for the 10 towns in with municipalized water within the 1875 and 1883 for which we need to construct investment data; Sheffield municipalized in 1887.

*Initial capital stock*

To apply the Perpetual Inventory Method, we need to pin down the initial capital stocks, but we lack such data. What we do instead is to use the value of loans outstanding in 1884, the first year in which it is disaggregated by type (i.e., water and sewerage). To address the issue of possible municipalisation of waterworks during this period, if an Urban District’s waterworks were initially privately owned but municipalised after 1884, we take the value of water loans outstanding in the first year after municipalisation. This is not unproblematic. The use of the stock of outstanding loans in 1884 as the anchor point for the construction of the capital stocks means that the level of the resulting series is determined by this choice. Insofar as 1884 stock is a function of the repayment schedules of the underlying loans or includes loans invested in stock that is no longer operational, these levels are mismeasured. For the purpose of our statistical analysis, however, this is not a problem as the level effects will be adjusted for by the fixed effects, but it is a potential problem for between-town comparisons of levels and therefore the data should be used with care for that purpose.

*The depreciation rate*

The final input to the Perpetual Inventory Method is the depreciation rate *δ*. From an economic point of view depreciation decline in the value of an asset over time due to use, wear and tear or obsolescence. The *current* spending on water and sewerage included maintenance of the capital stocks. Given that one might conjecture that the economic value of the capital stock was maintained over the period and therefore one should use a zero depreciation rate. We do so in the baseline. However, it is possible that the stock is not maintained or that parts of it becomes obsolete after some time. In that case, one should depreciate the stock. The OECD manual on the measurement of capital in Appendix 3 tabulate the depreciation rates used for different capital assets.^[[1]](#footnote-1)^ The one for water and sewerage capital is about 1 percent, but for other assets it is up to 3 percent. As a robustness check, we have constructed the two capital stock series with a depreciation rate of 3 percent and report in Appendix Table A4 **A2** and A6 **A4** results with these data. It makes little difference and so the choice of depreciation rate is not critical for the analysis.

# Control variables

In order to construct demographic and economic control variables for our 16 Urban Districts, we created a Geographical Information Systems (GIS) dataset of boundaries for the 16 Urban Districts in 1911.^[[2]](#footnote-2)^ This was augmented to reflect historical boundary changes to the 16 Urban Districts between 1870 and 1911. These boundaries were then used to identify sets of quasi-parish units that corresponded most closely to each of the Urban Districts for each census year. Individual-level census data were extracted from the Integrated MicroCensus (I-CeM) database for each of these quasi-parish units (into which the I-CeM data are already organised), and combining these with the GIS data, we constructed variables for each Urban District, for each decade following the census.^[[3]](#footnote-3)^ These variables were: measures of the age structure, sex ratio, and workers per capita employed in textiles, manufacturing and waterworks. The tax base per capita was extracted from the *Local Taxation Returns*, by dividing the ‘assessable value’ of all property in an Urban District by its population*.* A dummy equal to one if an Urban District had a municipal waterworks was constructed from parliamentary sources.^[[4]](#footnote-4)^

References

OECE, 2011. Measuring Capital. OECD Manual (OECD, Paris). Available <https://www.oecd.org/sdd/na/1876369.pdf> (Accessed October 2021).

Schürer, K. and E. Higgs., Integrated Census Microdata (I-CeM); 1851–1911 [computer file]. Colchester, Essex: UK Data Archive [distributor] (2014), SN: 7481. Available: <http://dx.doi.org/10.5255/UKDA-SN-7481-1>.

1. OECD ‘Measuring Capital’. [↑](#footnote-ref-1)
2. This dataset was created by Max Satchell, Cambridge Group for the History of Population and Social Structure. [↑](#footnote-ref-2)
3. Interpolated to provide annual values. Schürer & Higgs. ‘Integrated Census Microdata’. [↑](#footnote-ref-3)
4. *Means by which drinkable water* (P.P. 1878-9, LXI); *Water undertakings* (P.P. 1914, LXXIX). [↑](#footnote-ref-4)
