# Supplementary material for: New perspectives on the contribution of sanitary investments to mortality decline in English cities, 1845–1909
Source: Econ Hist Rev. 2022 Sep 26;76(2):624–60. doi: 10.1111/ehr.13195 (PMC10952366; doi:10.1111/ehr.13195)
Supplement: Supplementary file 2 — Appendixes tables A1–A6 [file EHR-76-624-s003.docx]

Table A1. The populations of the largest 16 non-metropolitan urban districts in England and Wales and their corresponding registration districts, 1871-1901.

| *Town* | *Municipal- isation  of water* | *UD population 1871* | *RDs com- prising UDs, population 1871* | *RD population % of UD population* | | | |
| --- | --- | --- | --- | --- | --- | --- | --- |
|  |  |  |  | *1871* | *1881* | *1891* | *1901* |
| Liverpool | 1848 | 493,405 | 581,336 | 118 | 124 | 146 | 119 |
| Manchester | 1851 | 351,189 | 463,340 | 132 | 155 | 121 | 124 |
| Birmingham | 1875 (1876) | 343,787 | 444,636 | 129 | 136 | 132 | 143 |
| Leeds | 1852 | 259,212 | 274,753 | 106 | 106 | 105 | 105 |
| Sheffield | 1887 (1888) | 239,946 | 249,703 | 104 | 105 | 106 | 107 |
| Bristol | - | 182,696 | 244,618 | 134 | 141 | 147 | 100 |
| Bradford | 1854 | 147,101 | 257,713 | 175 | 160 | 158 | 128 |
| Nottingham | 1880 | 138,876 | 204,971 | 148 | 148 | 155 | 100 |
| Newcastle | - | 128,443 | 131,198 | 102 | 103 | 106 | 109 |
| Kingston upon Hull | 1843 | 125,758 | 136,513 | 109 | 107 | 193 | 106 |
| Salford | 1850 | 124,801 | 128,890 | 103 | 103 | 103 | 104 |
| Portsmouth | - | 113,569 | 113,595 | 100 | 100 | 100 | 100 |
| Sunderland | - | 98,278 | 112,643 | 115 | 120 | 121 | 124 |
| Leicester | 1878 | 95,220 | 95,220 | 100 | 100 | 100 | 100 |
| Norwich | - | 80,386 | 80,386 | 100 | 100 | 100 | 100 |
| Wolverhampton | 1869 | 68,291 | 136,053 | 199 | 192 | 187 | 183 |
| 16 towns |  | 2,990,958 | 3,655,568 | 122 | 126 | 129 | 116 |
| Population of all UDs (ex. London) | | | | n/a | 13,820,163 | 17,516,969 | 20,521,814 |
| 16 towns % of all UDs (ex. London) | | | | n/a | 25 | 23 | 24 |
| Population of UDs with population 100,000+ (ex. London) | | | | n/a | 3,882,692 | 5,371,455 | 6,953,654 |
| 16 towns as % of UDs with population 100,000+ (ex. London) | | | | 113 | 90 | 67 | 70 |

*Notes:* Municipalisation date is the date of the relevant act (and when it came into effect). Summary statistics were not reported for all urban districts by size for 1871 in the censuses. The proportion of the population in towns with populations of 100,000 or more that was in our sample was 113 % in 1871 because the Registrar-General reported mortality for the largest 17 towns, including towns with populations under 100,000 (see column 2).

*Sources:* 1881 Census vol. II (P.P. 1883, LXXIX); 1891 Census vol. II (P.P. 1893-4, CV); 1901 Census (P.P. 1902, CIX-CXXI); Water undertakings (P.P. 1914, LXXIX).

Table A2. Extra regression results for all-cause mortality (1880-1909) and water, sewerage capital and the interaction between them from the *Local Taxation Returns* sample

|  | (1)^a^ | (2) | (3) | (4) | (5)^a^ |
| --- | --- | --- | --- | --- | --- |
| *Dependent variable:* | *All-cause mortality rate per 1000 population* | | | | |
|  |  |  |  |  |  |
| Water capital (WC) t-1 | -0.087 | 0.044 | 0.027 | 0.098 | 0.055 |
|  | (-1.17) | (0.38) | (0.23) | (1.29) | (0.85) |
| Sewerage capital (SC) t-1 | -0.21** | -0.72*** | -0.20 | -0.33** | -0.16*** |
|  | (-2.85) | (-6.35) | (-1.73) | (-2.37) | (-6.05) |
| WC×SC interaction t-1 |  | -0.57*** | -0.23*** | -0.24** | -0.28** |
|  |  | (-4.12) | (-3.38) | (-2.41) | (-2.62) |
| Tax base | 0.46*** |  | 0.41** | 0.12 | 0.33** |
|  | (3.62) |  | (2.80) | (0.66) | (2.40) |
| Population growth | 0.046** |  | -0.038 | 0.039 | 0.076** |
|  | (2.32) |  | (-1.06) | (1.47) | (2.24) |
| Female | -0.41 |  | -0.30 | 0.71 | -0.58*** |
|  | (-1.40) |  | (-1.33) | (1.17) | (-4.44) |
| Aged 0 to 14 | -1.09** |  | 0.37 | 0.88 | -0.87*** |
|  | (-2.99) |  | (1.30) | (1.61) | (-3.39) |
| Aged 15 to 44 | -0.52** |  | 0.12 | 0.49 | -0.23 |
|  | (-2.57) |  | (0.63) | (1.17) | (-1.02) |
| Birth rate | 0.76*** |  | 0.59*** | 0.58*** | 0.58*** |
|  | (4.93) |  | (3.36) | (4.68) | (4.05) |
| Manufacturing employment | 0.018 |  | -0.20** | 1.50** | 0.30 |
|  | (0.055) |  | (-2.49) | (2.77) | (0.68) |
| Textiles employment | -0.21 |  | 0.26* | -1.29 | -0.14 |
|  | (-0.36) |  | (2.18) | (-1.60) | (-0.38) |
|  |  |  |  |  |  |
| Observations | 63 | 63 | 63 | 63 | 63 |
| R-squared | 0.957 | 0.473 | 0.868 | 0.893 | 0.975 |
| Towns | 11 | 11 | 11 | 11 | 11 |
| Town FE | YES | NO | NO | YES | YES |
| Time FE | YES | NO | NO | NO | YES |
| Controls | YES | NO | YES | YES | YES |
| Method | OLS | OLS | OLS | OLS | OLS |
| Period | 1880-1909 | 1880-1909 | 1880-1909 | 1880-1909 | 1880-1909 |
| Std errors | clustered | clustered | clustered | clustered | clustered |
| P-value (Water)^b^ | 0.22 | 0.69 | 0.82 | 0.20 | 0.52 |
| P-value (Sewers)^b^ | 0.024 | 0.0040 | 0.23 | 0.041 | 0.011 |
| P-value (joint)^b^ | 0.040 | 0.18 | 0.20 | 0.30 | 0.021 |
| P-value (inter)^b^ |  | 0.059 | 0.14 | 0.20 | 0.21 |

*Notes*: All coefficients are standardised. Standard errors are clustered at the town level; t-statistics given in parentheses. R^2^ calculated for within variation. Regressions are weighted by average population over the period. All variables are measured at the Urban District (UD) level. Sample includes only town-year observations where a town’s water supply is municipally owned (and its expenditure therefore appears in its public accounts). Columns (2) to (5) progressively add controls and fixed effects to the specification with the interaction between water and sewerage capital. a. Replicates the baseline results from Table 4 with controls shown. b. The P-values refer to tests for significance of water capital, sewerage capital, the interaction of the two, and the joint significance of all of these based on a Wild bootstrap algorithm that clusters the standard errors at the town level. *** p<0.01, ** p<0.05, * p<0.1

Table A3. Extra regression results for all-cause mortality (1880-1909) and water and sewerage capital from the *Local Taxation Returns* sample

|  | *(1)* | *(2)* | *(3)* | *(4)* | *(5)* | *(6)* |
| --- | --- | --- | --- | --- | --- | --- |
| *Dependent variable:* | *All-cause mortality rate per 1000 population* | | | | | |
|  |  |  |  |  |  |  |
| Water capital (WC) t-1 | -0.092 | 0.076* | -0.12 | 0.036 | -0.13 | 0.032 |
|  | (-1.22) | (1.86) | (-1.10) | (0.40) | (-1.24) | (0.38) |
| Sewerage capital (SC) t-1 | -0.22* | -0.18*** | -0.16* | -0.15*** | -0.23* | -0.13** |
|  | (-2.00) | (-4.08) | (-2.10) | (-4.26) | (-1.98) | (-2.90) |
| WC×SC interaction t-1 |  | -0.37*** |  | -0.26* |  | -0.29** |
|  |  | (-6.74) |  | (-2.01) |  | (-2.74) |
|  |  |  |  |  |  |  |
| Observations | 57 | 57 | 63 | 63 | 63 | 63 |
| R-squared | 0.956 | 0.985 | 0.949 | 0.964 | 0.955 | 0.973 |
| Towns | 10 | 10 | 11 | 11 | 11 | 11 |
| Town FE | YES | YES | YES | YES | YES | YES |
| Time FE | YES | YES | YES | YES | YES | YES |
| Controls | YES | YES | YES | YES | YES | YES |
| Method | OLS | OLS | OLS | OLS | OLS | OLS |
| Period | 1880-1909 | 1880-1909 | 1880-1909 | 1880-1909 | 1880-1909 | 1880-1909 |
| Std errors | clustered | clustered | clustered | clustered | clustered | clustered |
| Without Salford | YES | YES | NO | NO | NO | NO |
| Without weighting | NO | NO | YES | YES | NO | NO |
| Winsorized^a^ | NO | NO | NO | NO | YES | YES |
| P-value (Water)^b^ | 0.16 | 0.16 | 0.36 | 0.78 | 0.29 | 0.77 |
| P-value (Sewers)^b^ | 0.048 | 0.068 | 0.20 | 0.024 | 0.070 | 0.080 |
| P-value (joint)^b^ | 0.071 | 0.027 | 0.33 | 0.23 | 0.033 | 0.0040 |
| P-value (inter)^b^ |  | 0.023 |  | 0.28 |  | 0.17 |
| Decline explained (Water)^c^ | 4.24 |  | 5.29 |  | 6.02 |  |
| Decline explained (Sewers)^c^ | 14.0 |  | 10.0 |  | 14.5 |  |
| Selection ratio^d^ | 0.93 |  | 0.72 |  | 1.13 |  |

*Notes*: Columns (1) and (2) report results without Salford; in columns (3) and (4) the data are not town-weighted; in columns (5) and (6) the data has been winsorized to eliminate the effect of outliers. All coefficients are standardised. Standard errors are clustered at the town level; t-statistics given in parentheses. R^2^ calculated for within variation. Regressions are weighted by average population over the period. All variables are measured at the Urban District (UD) level. Control variables included in all regressions are: Tax base, Population growth, Female, Age 0-14, Age 15-44, Crude birth rate, Manufacturing employment, and Textiles employment. Sample includes only town-year observations where a town’s water supply is municipally owned (and its expenditure therefore appears in its public accounts). a. The data for All-cause mortality and water capital are winsorized, i.e., the extreme values are replaced with 5 and 95 percentile values. b. The P-values refer to tests for significance of water capital, sewerage capital, the interaction of the two, and the joint significance of all of these based on a Wild bootstrap algorithm that clusters the standard errors at the town level. c. This is the percentage of the decline in the mortality rate that can be explained by the point estimate of the water and sewerage capital variables, respectively. d. The selection ratio reports the delta value representing the degree of selection on unobservables relative to observables that would be necessary to explain away the result related to sewerage capital as bias. *** p<0.01, ** p<0.05, * p<0.1

Table A4: Further regression results for all-cause mortality and water and sewerage capital from the *Local Taxation Returns* sample.

|  | *(1)* | *(2)* | *(3)* | *(4)* | *(5)* | *(6)* | *(7)* | *(8)* |
| --- | --- | --- | --- | --- | --- | --- | --- | --- |
| *Dependent variables* | *All-cause mortality rate* | | | | | | *Log All-cause mortality rate* | |
|  |  |  |  |  |  |  |  |  |
| Water capital (WC) t-1 | -0.084 | 0.060 | 0.066 | 0.045 | -0.049 | -0.086 | -0.011 | 0.0072 |
|  | (-1.00) | (1.09) | (0.83) | (0.66) | (-0.52) | (-0.91) | (-1.31) | (0.68) |
| Sewerage capital (SC) t-1 | -0.25** | -0.15** | -0.17** | -0.11*** | -0.13 | -0.055 | -0.039*** | -0.032*** |
|  | (-2.79) | (-3.04) | (-2.63) | (-3.95) | (-0.79) | (-0.34) | (-4.46) | (-7.18) |
| WC×SC interaction t-1 |  | -0.28* |  | -0.21** |  | -0.17 |  | -0.037* |
|  |  | (-2.20) |  | (-2.93) |  | (-1.13) |  | (-2.13) |
|  |  |  |  |  |  |  |  |  |
| Observations | 54 | 54 | 63 | 63 | 63 | 63 | 63 | 63 |
| R-squared | 0.954 | 0.971 | 0.959 | 0.972 | 0.988 | 0.990 | 0.971 | 0.980 |
| Number of id | 11 | 11 | 11 | 11 | 11 | 11 | 11 | 11 |
| Town FE | YES | YES | YES | YES | YES | YES | YES | YES |
| Time FE | YES | YES | YES | YES | YES | YES | YES | YES |
| Controls | YES | YES | YES | YES | YES | YES | YES | YES |
| Method | OLS | OLS | OLS | OLS | OLS | OLS | OLS | OLS |
| Period | 1885-1909 | 1885-1909 | 1880-1909 | 1880-1909 | 1880-1909 | 1880-1909 | 1880-1909 | 1880-1909 |
| Std errors | clustered | clustered | clustered | clustered | clustered | clustered | clustered | clustered |
| Depreciation rate | 0% | 0% | 3% | 3% | 0% | 0% | 0% | 0% |
| Town-specific trend | NO | NO | NO | NO | YES | YES | NO | NO |
| P-value (Water)^a^ | 0.33 | 0.42 | 0.46 | 0.56 | 0.59 | 0.39 | 0.16 | 0.64 |
| P-value (Sewers)^a^ | 0.0073 | 0.023 | 0.035 | 0.089 | 0.49 | 0.73 | 0.016 | 0.0080 |
| P-value (joint)^a^ | 0.031 | 0.068 | 0.086 | 0.028 | 0.62 | 0.46 | 0.011 | 0.018 |
| P-value (inter)^a^ |  | 0.21 |  | 0.22 |  | 0.41 |  | 0.25 |
| Decline explained (Water)^b^ | 3.85 |  | -3.02 |  | 2.24 |  |  |  |
| Decline explained (Sewers)^b^ | 16.0 |  | 10.9 |  | 7.98 |  |  |  |
| Selection ratio^c^ | 1.00 |  | 2.62 |  |  |  | 1.58 |  |

*Notes*: Columns (1) and (2) report results for the period (1885-1909) for which the LTRs report separate data for capital expenditures (loans) and current expenditures; columns (3) and (4) report results where we have applied a three percent depreciation rate in the construction of the capital stocks; columns (5) and (6) include a town-specific trend in the regression model; columns (7) and (8) show specifications where the dependent variable is the log of all-cause mortality rate. Dependent variable is all-cause mortality rate per 1,000 population or its logarithm. All coefficients are standardised except in the specification with the logarithm where the outcome variable it not. Standard errors are clustered at the town level; t-statistics given in parentheses. R^2^ calculated for within variation. Regressions are weighted by average population over the period. All variables are measured at the Urban District (UD) level. Control variables included in all regressions are: Tax base, Population growth, Female, Age 0-14, Age 15-44, Crude birth rate, Manufacturing employment, and Textiles employment. Sample includes only town-year observations where a town’s water supply is municipally owned (and its expenditure therefore appears in its public accounts). a. The P-values refer to tests for significance of water capital, sewerage capital, the interaction of the two, and the joint significance of all of these based on a Wild bootstrap algorithm that clusters the standard errors at the town level. b. This is the percentage of the decline in the mortality rate that can be explained by the point estimate of water and sewerage capital variables, respectively. c. The selection ratio reports the delta value representing the degree of selection on unobservables relative to observables that would be necessary to explain away the result related to sewerage capital as bias. Reported only for significant variables. *** p<0.01, ** p<0.05, * p<0.1

Table A5. Typhus and Typhoid mortality (1880-1909) and water and sewerage capital: regression results from the *Local Taxation Returns* sample.

|  | *(1)* | *(2)* | *(3)* | *(4)* |
| --- | --- | --- | --- | --- |
| *Dependent variables:* | *Typhus mortality rate* | | *Typhoid mortality rate* | |
|  |  |  |  |  |
| Water capital (WC) t-1 | -0.026 | 0.14 | 0.098 | 0.11 |
|  | (-0.18) | (0.95) | (1.37) | (1.12) |
| Sewerage capital (SC) t-1 | 0.17 | 0.23 | -0.12 | -0.12 |
|  | (0.84) | (1.45) | (-1.11) | (-1.00) |
| WC×SC interaction t-1 |  | -0.34* |  | -0.026 |
|  |  | (-1.91) |  | (-0.36) |
|  |  |  |  |  |
| Observations | 63 | 63 | 63 | 63 |
| R-squared | 0.843 | 0.868 | 0.775 | 0.775 |
| Towns | 11 | 11 | 11 | 11 |
| Town FE | YES | YES | YES | YES |
| Time FE | YES | YES | YES | YES |
| Controls | YES | YES | YES | YES |
| Method | OLS | OLS | OLS | OLS |
| Period | 1880-1909 | 1880-1909 | 1880-1909 | 1880-1909 |
| Std errors | Clustered | clustered | clustered | clustered |
| Unit | RD | RD | RD | RD |
| P-value (Water)^a^ | 0.89 | 0.51 | 0.14 | 0.20 |
| P-value (Sewers)^a^ | 0.63 | 0.39 | 0.38 | 0.47 |
| P-value (joint)^a^ | 0.87 | 0.74 | 0.44 | 0.64 |
| P-value (inter)^a^ |  | 0.31 |  | 0.70 |

*Notes*: Dependent variables are Typhus and the Typhoid mortality rate per 1,000 population, respectively. All coefficients are standardised. Standard errors are clustered at the town level; t-statistics given in parentheses. R^2^ calculated for within variation. Regressions are weighted by average population over the period. Outcome variables are measured at the Registration District (RD) level. Control variables included in all regressions are: Tax base (UD level), Population growth, Female, Age 0-14, Age 15-44, Crude birth rate, Manufacturing employment, and Textiles employment. Sample includes only town-year observations where a town’s water supply is municipally owned (and its expenditure therefore appears in its public accounts). a. The P-values refer to tests for significance of water capital, sewerage capital, the interaction of the two, and the joint significance of all of these based on a Wild bootstrap algorithm that clusters the standard errors at the town level. *** p<0.01, ** p<0.05, * p<0.1.

Table A6. Additional regression results for infant mortality (1845-1884) and water capital from the *Loans* sample.

|  | *(1)* | *(2)* | *(3)* | *(4)* | *(5)* | *(6)* |
| --- | --- | --- | --- | --- | --- | --- |
| *Dependent variable:* | *Infant mortality rate per 1000 births (IMR)* | | | | | *Log IMR* |
|  |  |  |  |  |  |  |
| Water capital, t-1 | -1.16** | -1.27*** | -1.73** | -1.06** | -0.60 | -1.20*** |
|  | (-8.81) | (-11.6) | (-3.93) | (-4.86) | (-1.03) | (-13.7) |
|  |  |  |  |  |  |  |
| Observations | 24 | 32 | 32 | 32 | 32 | 32 |
| R-squared | 0.865 | 0.838 | 0.842 | 0.839 | 0.851 | 0.855 |
| Number of id | 3 | 4 | 4 | 4 | 4 | 4 |
| Town FE | YES | YES | YES | YES | YES | YES |
| Time FE | YES | YES | YES | YES | YES | YES |
| Controls | YES | YES | YES | YES | YES | YES |
| Method | OLS | OLS | OLS | OLS | OLS | OLS |
| Period | 1845-1884 | 1845-1884 | 1845-1884 | 1845-1884 | 1845-1884 | 1845-1884 |
| Without Salford | YES | NO | NO | NO | NO | NO |
| Without weighting | NO | YES | NO | NO | NO | NO |
| Winzorised^a^ | NO | NO | YES | NO | NO | NO |
| Depreciation rate | 0% | 0% | 0% | 3% | 0% | 0% |
| Town-specific trend | NO | NO | NO | NO | YES | NO |
| P-value^b^ | 0.10 | 0.036 | 0.054 | 0.022 | 0.28 | 0.054 |
| Decline explained^c^ | 36.6 | 40.0 | 54.4 | 33.4 | 18.8 |  |
| Selection ratio^d^ | 1.12 | 1.25 | 1.17 | 1.66 |  | 1.46 |

*Notes:* Column (1) excludes Salford; in column (2), the data are not town-weighted; in column (3), the data are winzorised to eliminate the effect of outliers; in column (4), we apply a three percent depreciation rate in the construction of water capital; in column (5), we include town-specific time trends in the regression model; and in column (6), the dependent variable is the log of infant mortality. Infant mortality rate is per 1,000 births or the logarithm. Control variables are: Population growth and Crude birth rate. All coefficients are standardised except in the specification with the logarithm. t-statistics based on robust standard errors are given in parentheses. R^2^ calculated for within variation. Water capital and Population growth are measured at the Urban District (UD) level while the Infant mortality rate and Crude birth rate are measured at the Registration District (RD) level. a. The data for infant mortality and water capital are winsorized, i.e., the extreme values are replaced by the 5 and 95 percentile values. b. The P-values refer to test for significance of water capital based on a Wild bootstrap algorithm that clusters the standard errors at the town level. c. This is the percentage of the decline in the infant mortality rate that can be explained by the point estimate of water capital variable. d. The selection ratio reports the delta value representing the degree of selection on unobservables relative to observables that would be necessary to explain away the result related to water capital as bias. Reported only for significant variables. *** p<0.01, ** p<0.05, * p<0.1.
