# Supplementary material for: New perspectives on the contribution of sanitary investments to mortality decline in English cities, 1845–1909
Source: Econ Hist Rev. 2022 Sep 26;76(2):624–60. doi: 10.1111/ehr.13195 (PMC10952366; doi:10.1111/ehr.13195)
Supplement: Supplementary file 3 — Supporting Information [file EHR-76-624-s002.zip › deposit/~WRL1468.tmp]

Read me file

New perspectives on the contribution of sanitary investments to mortality decline in English cities, 1845-1909

The file provides an overview over replication data and code for this article. The code is written in Stata 16 and the data are provided in Stata “dta” format.

To execute the code, the copy the directory called “deposit” with its three subfolders “do”, “dta” and “output” to your computer.

The folder “do” contains the Stata do files with the code.

The folder “dta” contains the Stata “dta” files and has two sub-directories – “original data”, which contains the source data aggregated at the yearly, five year or 10 year frequency, and “working data” which contains the data with the standardized data and other transformations needed to run the regressions.

The folder “output” contains the Tables in Word and txt and the Figures generated when the various do files are executed.
