# Supplementary material for: New perspectives on the contribution of sanitary investments to mortality decline in English cities, 1845–1909
Source: Econ Hist Rev. 2022 Sep 26;76(2):624–60. doi: 10.1111/ehr.13195 (PMC10952366; doi:10.1111/ehr.13195)
Supplement: Supplementary file 3 — Supporting Information [file EHR-76-624-s002.zip › deposit/output/tables/table4.rtf]

Table 4.
	(1)	(2)	(3)	(4)	(5)	(6)	(7)	
VARIABLES	All-cause mortality rate	All-cause mortality rate	All-cause mortality rate	All-cause mortality rate	All-cause mortality rate	All-cause mortality rate	All-cause mortality rate	
								
Water capital (WC) t-1	-0.0099	-0.0019	-0.087	0.055			-0.37*	
	(-0.058)	(-0.018)	(-1.17)	(0.85)			(-1.90)	
Water capital (WC) (w. private) t-1					-0.058	0.023		
					(-0.85)	(0.29)		
Sewerage capital (SC) t-1	-0.72***	-0.40**	-0.21**	-0.16***	-0.18**	-0.060	-0.34***	
	(-3.98)	(-2.42)	(-2.85)	(-6.05)	(-2.15)	(-1.10)	(-2.81)	
WC x SC interaction t-1				-0.28**				
				(-2.62)				
WC x SC interaction  (w. private) t-1						-0.23***		
						(-3.80)		
Municipalised water					0.10	-0.062		
					(1.25)	(-0.99)		
Tax base		0.26	0.46***	0.33**	0.46***	0.33**	0.94**	
		(1.53)	(3.62)	(2.40)	(5.08)	(2.45)	(2.68)	
Population growth		0.032	0.046**	0.076**	0.038	0.054*	0.12***	
		(0.90)	(2.32)	(2.24)	(1.62)	(2.05)	(3.14)	
Female		0.90	-0.41	-0.58***	-0.13	-0.16	-0.96	
		(1.38)	(-1.40)	(-4.44)	(-0.58)	(-0.80)	(-1.68)	
Aged 0 to 14		0.83	-1.09**	-0.87***	-0.59**	-0.65***	-1.81***	
		(1.68)	(-2.99)	(-3.39)	(-2.75)	(-3.70)	(-4.93)	
Aged 15 to 44		0.42	-0.52**	-0.23	-0.21	-0.21	-1.09***	
		(0.98)	(-2.57)	(-1.02)	(-0.89)	(-1.01)	(-2.90)	
Birth rate		0.66***	0.76***	0.58***	0.60***	0.58***	0.98***	
		(4.34)	(4.93)	(4.05)	(4.52)	(6.91)	(4.80)	
Manufacturing employment		1.43**	0.018	0.30	0.20	0.52	-0.90	
		(2.54)	(0.055)	(0.68)	(0.92)	(1.58)	(-1.48)	
Textiles employment		-1.44	-0.21	-0.14	-0.29	-0.29	-0.088	
		(-1.79)	(-0.36)	(-0.38)	(-0.67)	(-1.17)	(-0.15)	
								
Observations	63	63	63	63	96	96	52	
R-squared	0.347	0.877	0.957	0.975	0.946	0.963	0.962	
City FE	NO	YES	YES	YES	YES	YES	YES	
Time FE	NO	NO	YES	YES	YES	YES	YES	
Controls	NO	YES	YES	YES	YES	YES	YES	
Method	OLS	OLS	OLS	OLS	OLS	OLS	LIML	
Period	1880-1909	1880-191	1880-1909	1880-1909	1880-1909	1880-1909	1885-1909	
Std errors	clustered	clustered	clustered	clustered	clustered	clustered	robust	
P-value (Water)	0.95	0.98	0.23	0.53	0.41	0.81	0.21	
P-value (Sewers)	0.024	0.17	0.029	0.0090	0.10	0.32	0.051	
P-value (joint)	0.052	0.24	0.042	0.020	0.21	0.0086	0.091	
P-value (inter)	-1			0.21		0.092		
Decline explained (Water)	0.46	0.090	4		2.67		17.1	
Decline explained (Sewers)	45.7	25.2	13.3		11.7		22	
Number of id		11	11	11	16	16	11	
K-P							4.75	
Selection ratio			1.11		0.97			
Robust t-statistics in parentheses
*** p<0.01, ** p<0.05, * p<0.1
