# Supplementary material for: New perspectives on the contribution of sanitary investments to mortality decline in English cities, 1845–1909
Source: Econ Hist Rev. 2022 Sep 26;76(2):624–60. doi: 10.1111/ehr.13195 (PMC10952366; doi:10.1111/ehr.13195)
Supplement: Supplementary file 3 — Supporting Information [file EHR-76-624-s002.zip › deposit/output/tables/table5.rtf]

Table 5.
	(1)	(2)	(3)	(4)	(5)	(6)	
VARIABLES	Diarrhoea mortality rate	Diarrhoea mortality rate	Diarrhoea mortality rate	Infant mortality rate	Infant mortality rate	Infant mortality rate	
							
Water capital (WC) t-1	-0.39	0.011	-0.091	-0.017	0.030	-0.027	
	(-1.39)	(0.060)	(-0.57)	(-0.071)	(0.22)	(-0.22)	
Sewerage capital (SC) t-1	-0.28	-0.068	-0.10	-0.66***	-0.32	-0.34	
	(-1.16)	(-0.28)	(-0.46)	(-4.94)	(-1.56)	(-1.71)	
WC x SC interaction t-1			0.20			0.11	
			(1.06)			(0.56)	
Tax base	1.30*	-0.31	-0.22	0.94**	0.15	0.20	
	(2.23)	(-1.01)	(-0.79)	(3.12)	(0.55)	(0.80)	
Population growth	0.29**	0.0065	-0.015	0.25**	0.075	0.064	
	(2.37)	(0.092)	(-0.20)	(2.40)	(1.70)	(1.29)	
Female	-3.38***	-1.36*	-1.24*	-0.094	-0.37	-0.30	
	(-3.18)	(-2.16)	(-1.86)	(-0.14)	(-0.74)	(-0.56)	
Aged 0 to 14	0.19	-0.34	-0.50	2.45***	0.20	0.11	
	(0.25)	(-0.55)	(-0.86)	(4.20)	(0.46)	(0.27)	
Aged 15 to 44	0.69	-0.43	-0.64	1.99***	0.31	0.19	
	(1.16)	(-0.97)	(-1.65)	(4.09)	(0.74)	(0.48)	
Birth rate	0.61**	-0.033	0.098	0.082	0.11	0.19	
	(2.96)	(-0.26)	(0.61)	(0.41)	(0.80)	(1.55)	
Manufacturing employment	-2.49**	-1.21	-1.41*	2.14***	1.08	0.97	
	(-2.44)	(-1.61)	(-1.88)	(3.52)	(1.17)	(1.04)	
Textiles employment	-1.50	1.29	1.24	-2.98**	-0.29	-0.32	
	(-0.98)	(1.59)	(1.40)	(-3.01)	(-0.39)	(-0.42)	
							
Observations	63	63	63	63	63	63	
R-squared	0.567	0.877	0.885	0.783	0.914	0.916	
Number of id	11	11	11	11	11	11	
Town FE	YES	YES	YES	YES	YES	YES	
Time FE	NO	YES	YES	NO	YES	YES	
Controls	YES	YES	YES	YES	YES	YES	
Method	OLS	OLS	OLS	OLS	OLS	OLS	
Period	1880-1909	1880-1909	1880-1909	1880-1909	1880-1909	1880-1909	
Std errors	clustered	clustered	clustered	clustered	clustered	clustered	
Unit	UD	UD	UD	UD	UD	UD	
P-value (Water)	0.75	0.97	0.75	0.95	0.88	0.84	
P-value (Sewers)	0.41	0.94	0.93	0.061	0.59	0.56	
P-value (joint)	0.76	0.99	0.97	0.057	0.61	0.73	
P-value (inter)	-1		0.50			0.66	
Robust t-statistics in parentheses
*** p<0.01, ** p<0.05, * p<0.1
