# Supplementary material for: New perspectives on the contribution of sanitary investments to mortality decline in English cities, 1845–1909
Source: Econ Hist Rev. 2022 Sep 26;76(2):624–60. doi: 10.1111/ehr.13195 (PMC10952366; doi:10.1111/ehr.13195)
Supplement: Supplementary file 3 — Supporting Information [file EHR-76-624-s002.zip › deposit/output/tables/table6.rtf]

Table 6.
	(1)	(2)	(3)	(4)	(5)	(6)	(7)	
VARIABLES	All-cause mortality rate	All-cause mortality rate	All-cause mortality rate	All-cause mortality rate	All-cause mortality rate	All-cause mortality rate	All-cause mortality rate	
								
Debt (square root)	-0.091	-0.087	-0.10	-0.33*	-0.30***	-0.60*	-0.40	
	(-1.59)	(-1.68)	(-1.17)	(-2.13)	(-2.96)	(-1.82)	(-0.69)	
								
Observations	94	48	48	61	77	61	48	
R-squared	0.937	0.980	0.957	0.927	0.960	0.949	0.950	
Number of id	16	16	16	16	16	16	16	
Town FE	YES	YES	YES	YES	YES	YES	YES	
Time FE	YES	YES	YES	YES	YES	YES	YES	
Controls	YES	YES	YES	YES	YES	YES	YES	
Method	OLS	OLS	OLS	OLS	OLS	LILM	LIML	
Period	1871-1900	1871-1900	1871-1900	1861-1900	1861-1910	1871-1910	1881-1910	
Std errors	clustered	clustered	clustered	clustered	clustered	Robust	Robust	
Unit	UD	UD	RD	RD	RD	RD	RD	
Frequency	five	Decenial	Decenial	Decenial	Decenial	Decenial	Decenial	
P-value (Loans)	0.12	0.16	0.27	0.060	0.00030	0.010	0.45	
Decline explained	11.6	9.52	11.2	35.3	26.6	52.7	34.6	
K-P F-statistics						6.77	3.81	
Selection ratio				0.35	0.51			
Robust t-statistics in parentheses
*** p<0.01, ** p<0.05, * p<0.1
