# Supplementary material for: New perspectives on the contribution of sanitary investments to mortality decline in English cities, 1845–1909
Source: Econ Hist Rev. 2022 Sep 26;76(2):624–60. doi: 10.1111/ehr.13195 (PMC10952366; doi:10.1111/ehr.13195)
Supplement: Supplementary file 3 — Supporting Information [file EHR-76-624-s002.zip › deposit/output/tables/table7.rtf]

Table 7
	(1)	(2)	(3)	(4)	(5)	(6)	(7)	
VARIABLES	Infant mortality rate	Infant mortality rate	Infant mortality rate	Infant mortality rate	Infant mortality rate	Infant mortality rate incl 1838-44	Infant mortality rate	
								
Water capital, t-1	-1.12***	-1.71***	-1.25***	-1.23***	-1.38***	-1.29***	-1.19**	
	(-4.43)	(-14.6)	(-10.1)	(-8.93)	(-4.85)	(-14.3)	(-3.34)	
Crude birth rate				-0.26	-0.25		0.20	
				(-1.99)	(-1.69)		(1.30)	
Population growth				0.065	0.076	0.080	0.11	
				(0.59)	(0.94)	(0.64)	(0.79)	
Crude bith rate incl 1838-44						-0.23		
						(-1.54)		
Constant	0.045	-0.22**	-0.24**	-0.27**		-0.33***	-0.14	
	(0.28)	(-4.17)	(-3.44)	(-3.60)		(-7.98)	(-1.76)	
								
Observations	32	32	32	32	32	36	24	
R-squared	0.211	0.501	0.814	0.845	0.843	0.823	0.862	
Town FE	NO	YES	YES	YES	YES	YES	YES	
Time FE	NO	NO	YES	YES	YES	YES	YES	
Controls	NO	NO	NO	YES	YES	YES	YES	
Method	OLS	OLS	OLS	OLS	IV	OLS	OLS	
Period	1845-1884	1845-1884	1845-1884	1845-1884	1845-1884	1840-1884	1855-	
P-value	0.36	0.18	0.17	0.061	0.034	0.033	0.053	
Decline explained (Water)	35.4	54	39.5	38.8	43.6	40.6	37.6	
Number of id		4	4	4	4	4	4	
Selection ratio				1.40		1.21	1.70	
K-P					90.1			
Robust t-statistics in parentheses
*** p<0.01, ** p<0.05, * p<0.1
