# Supplementary material for: New perspectives on the contribution of sanitary investments to mortality decline in English cities, 1845–1909
Source: Econ Hist Rev. 2022 Sep 26;76(2):624–60. doi: 10.1111/ehr.13195 (PMC10952366; doi:10.1111/ehr.13195)
Supplement: Supplementary file 3 — Supporting Information [file EHR-76-624-s002.zip › deposit/output/tables/table8.rtf]

Table 8
	(1)	(2)	(3)	(4)	(5)	(6)	(7)	(8)	(9)	(10)	(11)	(12)	(13)	(14)	(15)	
VARIABLES	Infant mortality rate	Infant mortality rate	Infant mortality rate	Infant mortality rate	Infant mortality rate	Infant mortality rate	Infant mortality rate	Infant mortality rate	Infant mortality rate	Infant mortality rate	Infant mortality rate	Infant mortality rate	Infant mortality rate	Infant mortality rate	Infant mortality rate	
																
Water capital, t-1	-1.31*	-1.23***	-0.40	-0.45	-0.099						-2.19*	-1.38***	-0.53*	-0.44**	0.045	
	(-2.37)	(-8.93)	(-1.10)	(-1.76)	(-0.75)						(-2.17)	(-4.85)	(-1.73)	(-2.18)	(0.17)	
Water capital, t-2						-2.04***	-1.94***	-0.47	-0.34	0.022						
						(-6.86)	(-8.32)	(-1.97)	(-1.83)	(0.12)						
Crude birth rate	-0.37**	-0.26	-0.18	0.0014	0.067	-0.38*	-0.24	-0.20	0.054	0.078	-0.33**	-0.25	-0.18	0.0032	0.084	
	(-3.81)	(-1.99)	(-0.69)	(0.0075)	(0.65)	(-2.42)	(-1.46)	(-0.74)	(0.28)	(0.83)	(-2.31)	(-1.69)	(-0.77)	(0.026)	(0.85)	
Population growth	0.037	0.065	-0.023	0.0073	-0.014	-0.055	-0.071	-0.044	-0.046	-0.037	-0.057	0.076	0.015	0.0032	-0.043	
	(0.23)	(0.59)	(-0.14)	(0.058)	(-0.23)	(-0.39)	(-0.83)	(-0.49)	(-0.54)	(-0.67)	(-0.50)	(0.94)	(0.13)	(0.038)	(-0.74)	
Constant	0.41	-0.27**	0.35	-0.65*	-0.98***	-0.12	-0.91***	0.34	-0.75*	-1.11***						
	(0.90)	(-3.60)	(0.69)	(-2.78)	(-6.90)	(-0.32)	(-8.67)	(0.80)	(-3.02)	(-6.45)						
																
Observations	24	32	40	52	28	24	32	40	52	28	24	32	40	52	28	
R-squared	0.661	0.845	0.759	0.824	0.790	0.700	0.857	0.767	0.804	0.785	0.635	0.843	0.754	0.824	0.779	
Number of id	4	4	4	4	4	4	4	4	4	4	4	4	4	4	4	
Town FE	YES	YES	YES	YES	YES	YES	YES	YES	YES	YES	YES	YES	YES	YES	YES	
Time FE	YES	YES	YES	YES	YES	YES	YES	YES	YES	YES	YES	YES	YES	YES	YES	
Controls	YES	YES	YES	YES	YES	YES	YES	YES	YES	YES	YES	YES	YES	YES	YES	
Method	OLS	OLS	OLS	OLS	OLS	OLS	OLS	OLS	OLS	OLS	LIML	LIML	LIML	LIML	LIML	
Period	1845-1874	1845-1884	1845-1894	1845-1909	1875-1909	1845-1874	1845-1884	1845-1894	1845-1909	1875-1909	1845-1874	1845-1884	1845-1894	1845-1909	1875-1909	
P-value	0.23	0.062	0.56	0.44	0.40	0.057	0.054	0.41	0.38	0.87	0.14	0.034	0.44	0.38	0.87	
Selection ratio	0.66	1.40				0.50	0.99									
K-P											25	90.1	60	94.8	10.8	
Robust t-statistics in parentheses
*** p<0.01, ** p<0.05, * p<0.1
