# Supplementary material for: New perspectives on the contribution of sanitary investments to mortality decline in English cities, 1845–1909
Source: Econ Hist Rev. 2022 Sep 26;76(2):624–60. doi: 10.1111/ehr.13195 (PMC10952366; doi:10.1111/ehr.13195)
Supplement: Supplementary file 3 — Supporting Information [file EHR-76-624-s002.zip › deposit/output/tables/table9.rtf]

Table 9
	(1)	(2)	(3)	(4)	(5)	(6)	(7)	(8)	
VARIABLES	Early childhood mortality rate	Early childhood mortality rate	Crude death rate	Crude death rate	Life expectancy at birth	Life expectancy at birth	Life expectancy at age 15	Life expectancy at age 15	
									
Water capital, t-1	-0.51**	-0.47**	-0.41*	-0.44***	0.58**	0.64***	0.11	0.17	
	(-3.64)	(-2.46)	(-2.43)	(-3.09)	(3.76)	(3.42)	(0.40)	(0.75)	
Crude birth rate	0.25**	0.25***	0.38***	0.38***	-0.13	-0.13*	-0.30***	-0.31***	
	(4.71)	(3.73)	(7.49)	(6.44)	(-2.15)	(-1.76)	(-8.39)	(-3.32)	
Population growth	0.037	0.033	-0.063	-0.060	0.057	0.053	0.18	0.18*	
	(0.60)	(0.52)	(-0.56)	(-0.89)	(0.59)	(0.81)	(1.16)	(2.03)	
Constant	-0.65***		0.047		0.54**		0.049		
	(-11.5)		(0.82)		(5.42)		(0.27)		
									
Observations	32	32	32	32	32	32	32	32	
R-squared	0.926	0.925	0.921	0.921	0.912	0.912	0.831	0.830	
Number of id	4	4	4	4	4	4	4	4	
Town FE	YES	YES	YES	YES	YES	YES	YES	YES	
Time FE	YES	YES	YES	YES	YES	YES	YES	YES	
Controls	YES	YES	YES	YES	YES	YES	YES	YES	
Method	OLS	LIML	OLS	LIML	OLS	LIML	OLS	LIML	
Period	1845-1884	1845-1884	1845-1884	1845-1884	1845-1884	1845-1884	1845-1884	1845-1884	
P-value	0.17	0.092	0.27	0.34	0.19	0.096	0.72	0.82	
Decline explained (Water)	16.1	14.7	13	14	18.3	20	3.47	5.48	
Selection ratio	1.30								
K-P		90.1		90.1		90.1		90.1	
Robust t-statistics in parentheses
*** p<0.01, ** p<0.05, * p<0.1
