# Supplementary material for: New perspectives on the contribution of sanitary investments to mortality decline in English cities, 1845–1909
Source: Econ Hist Rev. 2022 Sep 26;76(2):624–60. doi: 10.1111/ehr.13195 (PMC10952366; doi:10.1111/ehr.13195)
Supplement: Supplementary file 3 — Supporting Information [file EHR-76-624-s002.zip › deposit/output/tables/tableA3.rtf]

Table A3.
	(1)	(2)	(3)	(4)	(5)	(6)	
VARIABLES	All-cause mortality rate	All-cause mortality rate	All-cause mortality rate	All-cause mortality rate	All-cause mortality rate	All-cause mortality rate	
							
Water capital (WC) t-1	-0.092	0.076*	-0.12	0.036	-0.13	0.032	
	(-1.22)	(1.86)	(-1.10)	(0.40)	(-1.24)	(0.38)	
Sewerage capital (SC) t-1	-0.22*	-0.18***	-0.16*	-0.15***	-0.23*	-0.13**	
	(-2.00)	(-4.08)	(-2.10)	(-4.26)	(-1.98)	(-2.90)	
WC x SC interaction t-1		-0.37***		-0.26*		-0.29**	
		(-6.74)		(-2.01)		(-2.74)	
Tax base	0.45*	0.29	0.47***	0.33*	0.51***	0.34*	
	(2.18)	(1.57)	(5.65)	(2.18)	(3.34)	(2.19)	
Population growth	0.051*	0.097***	0.043*	0.066	0.044*	0.068*	
	(1.98)	(3.66)	(1.99)	(1.50)	(1.91)	(2.11)	
Female	-0.38	-0.63***	-0.52	-0.66***	-0.53*	-0.70***	
	(-1.12)	(-4.11)	(-1.68)	(-3.27)	(-1.91)	(-4.90)	
Aged 0 to 14	-1.05**	-0.71***	-1.06**	-0.97***	-1.35**	-1.15***	
	(-2.74)	(-4.07)	(-2.83)	(-3.17)	(-2.94)	(-3.91)	
Aged 15 to 44	-0.48**	-0.077	-0.59**	-0.38	-0.72**	-0.42	
	(-2.39)	(-0.50)	(-2.33)	(-1.24)	(-2.74)	(-1.72)	
Birth rate	0.75***	0.50***	0.74***	0.62***	0.75***	0.58***	
	(4.68)	(4.66)	(5.57)	(4.55)	(3.96)	(3.57)	
Manufacturing employment	0.12	0.61	-0.16	0.037	-0.0036	0.27	
	(0.40)	(1.82)	(-0.50)	(0.075)	(-0.012)	(0.63)	
Textiles employment	-0.22	-0.22	-0.26	-0.13	-0.060	0.091	
	(-0.27)	(-0.58)	(-0.46)	(-0.35)	(-0.11)	(0.29)	
							
Observations	57	57	63	63	63	63	
R-squared	0.956	0.985	0.949	0.964	0.955	0.973	
Number of id	10	10	11	11	11	11	
Town FE	YES	YES	YES	YES	YES	YES	
Time FE	YES	YES	YES	YES	YES	YES	
Controls	YES	YES	YES	YES	YES	YES	
Method	OLS	OLS	OLS	OLS	OLS	OLS	
Period	1880-1909	1880-1909	1880-1909	1880-1909	1880-1909	1880-1909	
Std errors	clustered	clustered	clustered	clustered	clustered	clustered	
Without Salford	YES	YES	NO	NO	NO	NO	
Without weighting	NO	NO	YES	YES	NO	NO	
Winsoried	NO	NO	NO	NO	YES	YES	
P-value (Water)	0.15	0.17	0.36	0.78	0.29	0.77	
P-value (Sewers)	0.046	0.070	0.21	0.023	0.068	0.080	
P-value (joint)	0.071	0.026	0.32	0.23	0.035	0.0026	
P-value (inter)	-1	0.023		0.28		0.18	
Decline explained (Water)	4.24		5.29		6.02		
Decline explained (Sewers)	14		10		14.5		
Selection ratio	0.93		0.72		1.13		
Robust t-statistics in parentheses
*** p<0.01, ** p<0.05, * p<0.1
