# Supplementary material for: New perspectives on the contribution of sanitary investments to mortality decline in English cities, 1845–1909
Source: Econ Hist Rev. 2022 Sep 26;76(2):624–60. doi: 10.1111/ehr.13195 (PMC10952366; doi:10.1111/ehr.13195)
Supplement: Supplementary file 3 — Supporting Information [file EHR-76-624-s002.zip › deposit/output/tables/tableA5.rtf]

Table A5.
	(1)	(2)	(3)	(4)	
VARIABLES	Typhus mortality rate	Typhus mortality rate	Typhoid mortality rate	Typhoid mortality rate	
					
Water capital (WC) t-1	-0.026	0.14	0.098	0.11	
	(-0.18)	(0.95)	(1.37)	(1.12)	
Sewerage capital (SC) t-1	0.17	0.23	-0.12	-0.12	
	(0.84)	(1.45)	(-1.11)	(-1.00)	
WC x SC interaction t-1		-0.34*		-0.026	
		(-1.91)		(-0.36)	
Tax base	-0.88	-1.03	-0.19	-0.20	
	(-1.48)	(-1.64)	(-0.54)	(-0.56)	
Population growth	-0.087	-0.052	0.063	0.066	
	(-0.97)	(-0.54)	(1.45)	(1.52)	
Female	0.26	0.052	-0.51	-0.52	
	(0.35)	(0.068)	(-0.94)	(-0.96)	
Aged 0 to 14	1.28**	1.54***	-1.27**	-1.25**	
	(2.36)	(3.38)	(-2.29)	(-2.30)	
Aged 15 to 44	3.14***	3.49***	-0.55	-0.52	
	(4.06)	(4.12)	(-0.97)	(-0.94)	
Birth rate	-0.32	-0.54**	0.25	0.24	
	(-1.59)	(-3.05)	(0.86)	(0.73)	
Manufacturing employment	1.39*	1.72	0.017	0.042	
	(1.83)	(1.79)	(0.029)	(0.073)	
Textiles employment	1.20	1.30	1.21	1.22	
	(1.14)	(1.35)	(1.54)	(1.53)	
					
Observations	63	63	63	63	
R-squared	0.843	0.868	0.775	0.775	
Number of id	11	11	11	11	
City FE	YES	YES	YES	YES	
Time FE	YES	YES	YES	YES	
Controls	YES	YES	YES	YES	
Method	OLS	OLS	OLS	OLS	
Period	1875-1909	1875-1909	1875-1909	1875-1909	
Std errors	clustered	clustered	clustered	clustered	
Unit	RD	RD	RD	RD	
P-value (Water)	0.89	0.53	0.14	0.22	
P-value (Sewers)	0.66	0.35	0.40	0.42	
P-value (joint)	0.87	0.75	0.44	0.64	
P-value (inter)	-1	0.30		0.71	
Robust t-statistics in parentheses
*** p<0.01, ** p<0.05, * p<0.1
