# Supplementary material for: New perspectives on the contribution of sanitary investments to mortality decline in English cities, 1845–1909
Source: Econ Hist Rev. 2022 Sep 26;76(2):624–60. doi: 10.1111/ehr.13195 (PMC10952366; doi:10.1111/ehr.13195)
Supplement: Supplementary file 3 — Supporting Information [file EHR-76-624-s002.zip › deposit/output/tables/tableA6.rtf]

Table A6
	(1)	(2)	(3)	(4)	(5)	(6)	
VARIABLES	Infant mortality rate	Infant mortality rate	Infant mortality rate	Infant mortality rate	Infant mortality rate	Log infant mortality rate	
							
Water capital, t-1	-1.16**	-1.27***	-1.73**		-0.60	-1.20***	
	(-8.81)	(-11.6)	(-3.93)		(-1.03)	(-13.7)	
Water capital (3%), t-1				-1.06**			
				(-4.86)			
Crude birth rate	-0.33	-0.18	-0.23	-0.26	-0.23	-0.23	
	(-2.58)	(-1.41)	(-2.20)	(-2.15)	(-1.17)	(-2.07)	
Population growth	0.088	0.057	-0.069	0.083	-0.0057	0.042	
	(0.73)	(0.50)	(-0.93)	(0.74)	(-0.036)	(0.43)	
Constant	-0.16	-0.55***	-0.74*	-0.20**	12.4	-0.27*	
	(-1.94)	(-6.64)	(-3.00)	(-4.30)	(0.93)	(-3.04)	
							
Observations	24	32	32	32	32	32	
R-squared	0.865	0.838	0.842	0.839	0.851	0.855	
Number of id	3	4	4	4	4	4	
Town FE	YES	YES	YES	YES	YES	YES	
Time FE	YES	YES	YES	YES	YES	YES	
Controls	YES	YES	YES	YES	YES	YES	
Method	OLS	OLS	OLS	OLS	OLS	OLS	
Period	1845-1884	1845-1884	1845-1884	1845-1884	1845-1884	1845-1884	
Without Salford	YES	NO	NO	NO	NO	NO	
Without weighting	NO	YES	NO	NO	NO	NO	
Winzorised	NO	NO	YES	NO	NO	NO	
Depreciation rate	0%	0%	0%	3%	0%	0%	
Town-specific trend	NO	NO	NO	NO	YES	NO	
P-value	0.10	0.034	0.054	0.035	0.28	0.051	
Decline explained (Water)	36.6	40	54.7	33.4	18.8		
Selection ratio	1.12	1.25	1.17	1.66	0.11	1.46	
Robust t-statistics in parentheses
*** p<0.01, ** p<0.05, * p<0.1
