# Supplementary material for: New perspectives on the contribution of sanitary investments to mortality decline in English cities, 1845–1909
Source: Econ Hist Rev. 2022 Sep 26;76(2):624–60. doi: 10.1111/ehr.13195 (PMC10952366; doi:10.1111/ehr.13195)
Supplement: Supplementary file 3 — Supporting Information [file EHR-76-624-s002.zip › deposit/Read me file.docx]

**Read me file**

New perspectives on the contribution of sanitary investments to mortality decline in English cities, 1845-1909

The file provides an overview over the replication data and code for this article. The code is written in Stata 16 and the data are provided as Stata Datasets in “dta” format.

To execute the code, copy the directory called “deposit” with its three subfolders “do”, “dta” and “output” to your computer and adjust the root director in the do files to match you local settings.

The folder “do” contains the Stata do files with the code.

The folder “dta” contains the Stata “dta” files and has two sub-directories – “original data”, which contains the source data aggregated at the yearly, quinquennial or decadal frequency, and “working data” which contains the files with the standardized data and other transformations needed to run the regressions.

The folder “output” stores the tables in Word and txt and the figures generated when the various do files are executed. The word documents needs some manual editing to match the tables in the paper in terms of style and it is easier to see them via the “dir : seeout” link in the output window.

**The do files**

Each table and figure has its own do file named tableX or figureX as appropriate. These reproduce the relevant table or figure reported in the text, the appendix or in the supplementary appendix. For the regression tables, the outreg2 procedure outputs the results. This can be switched off if it interferes with the bootstrap calculations.

The two do files called

- prepare regression data LOANS.do
- prepare regression data LRT.do

contain the code to transform the original data based on the LRT or the Loans sample into the Stata Datasets used as input to the regression analysis. The outputs from these do files are saved in the dta/working data directory. The directory already contains these files, so you do not need to run these files, but can do so.

The do files uses various packages that may not be installed already. The list is

| wtmean | fttools |
| --- | --- |
| boottest | eststo |
| psacalc | outreg2 |
| interflex | winsor2 |
| reghdfe | xtivreg2 |

**The dta files**

The Stata Datasets are stored in the folder “original data”. Table 1 provides an overview of what they contain and where they are used.

Table 1. Overview of the Stata Datasets

| Name | Content | Frequency | Units | Period | Use |
| --- | --- | --- | --- | --- | --- |
| LTR 5 original | Water and sewerage capital, mortality, controls | quinquennial | 16 UDs | 1870-1909 | T3, T4, T5, F9, A2, A3, A4, A5.^a^ |
| loans 5 original | Water capital, mortality, controls | quinquennial | 4 UDs, RDs | 1835-1909 | T3, T7, T8, T9, A6.^a^ |
| LTR 10 original | Water and sewerage capital, mortality, controls | Decadal | 16 UDs, RDs | 1860-1910 | T6.^a^ |
| tables1 | Fiscal data and controls | Yearly | 16 UDs | 1883-1901 | TS1 |
| figure2 | Mortality and fiscal data | Yearly | England and Wales | 1848-1910 | F2 |
| figure3 | Water and sewerage capital | Yearly | 16 UDs | 1872-1910 | F3 |
| figure4 | Water loans and capital | Yearly | 4 UDs | 1835-1910 | F4 |
| figure6a | Mortality data | Yearly | 16 UDs (ag) | 1870-1911 | F6a |
| figure6b_d | Mortality data | Yearly | 16 UDs | 1870-1911 | F6b to F6d |
| figure7 | Mortality data | Yearly | 16 RDs (ag) | 1870-1911 | F7a to F7b |
| figure8 | Mortality data | Yearly | 4 RDs | 1847-1909 | F8 |
| figureS1_S2 | Water and sewerage capital expenditure | Yearly | 16 UDs | 1883-1900 | FS1, FS2 |

Note: T = Table, F = Figure, UD = Urban District , RD = Registration District (associated with the UD), ag = aggregate. a. The three Stata Datasets are used to created the output in these tables and figures after they have been transformed by “prepare regression data LOANS.do” or “prepare regression data LRT.do”.
