# Supplementary figures and images for: New perspectives on the contribution of sanitary investments to mortality decline in English cities, 1845–1909
Source: Econ Hist Rev. 2022 Sep 26;76(2):624–60. doi: 10.1111/ehr.13195 (PMC10952366; doi:10.1111/ehr.13195)

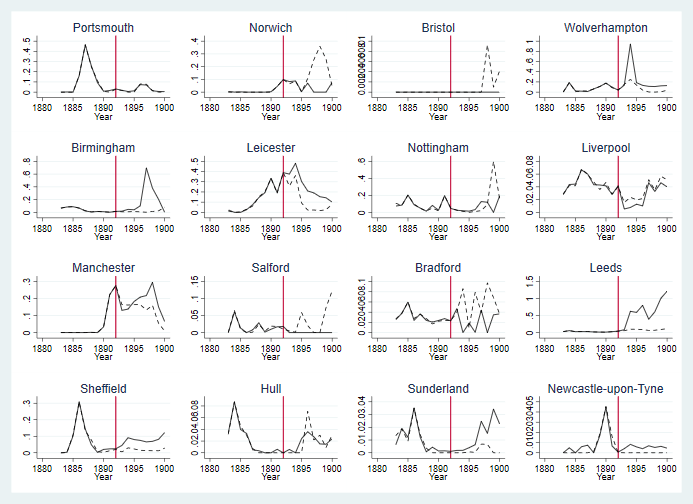

Supplement: Supplementary file 3 — Supporting Information [file EHR-76-624-s002.zip › deposit/output/figures/Figure_prediction_sewers_all.png]

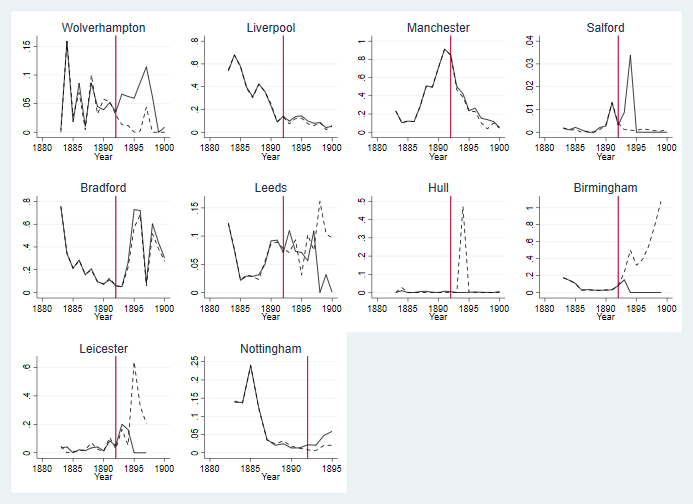

Supplement: Supplementary file 3 — Supporting Information [file EHR-76-624-s002.zip › deposit/output/figures/Figure_prediction_water_all.png]

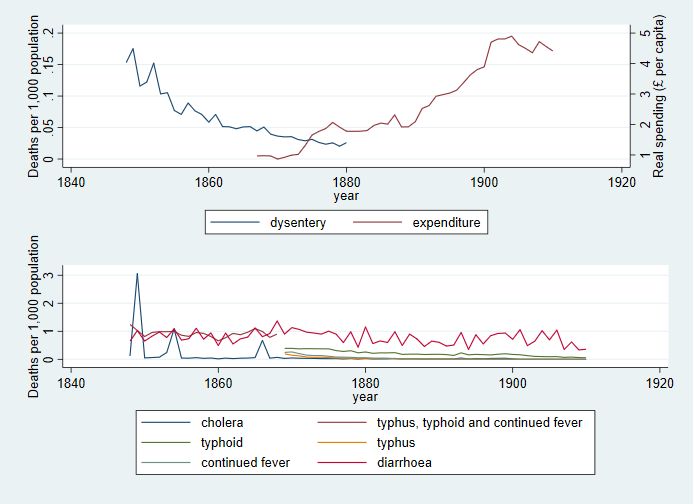

Supplement: Supplementary file 3 — Supporting Information [file EHR-76-624-s002.zip › deposit/output/figures/Figure2.png]

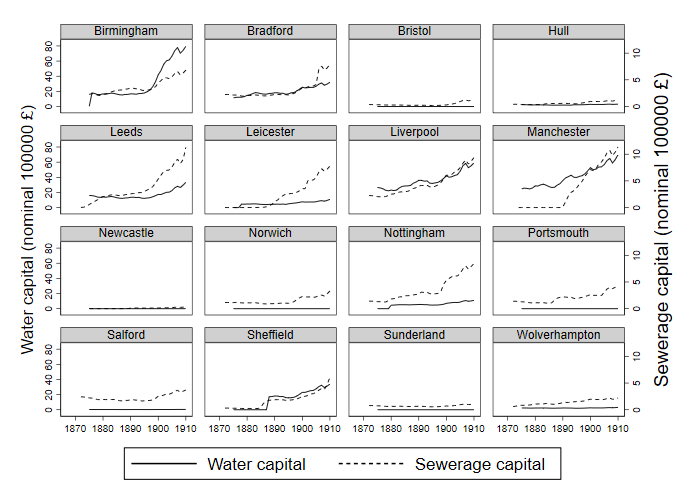

Supplement: Supplementary file 3 — Supporting Information [file EHR-76-624-s002.zip › deposit/output/figures/Figure3.png]

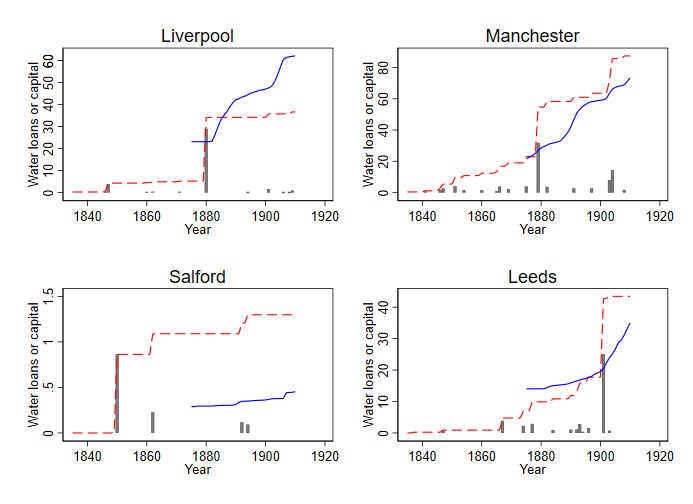

Supplement: Supplementary file 3 — Supporting Information [file EHR-76-624-s002.zip › deposit/output/figures/Figure4.png]

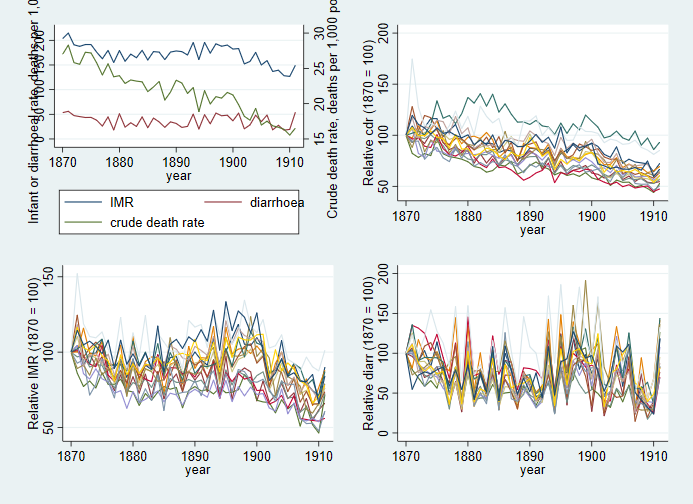

Supplement: Supplementary file 3 — Supporting Information [file EHR-76-624-s002.zip › deposit/output/figures/Figure6.png]

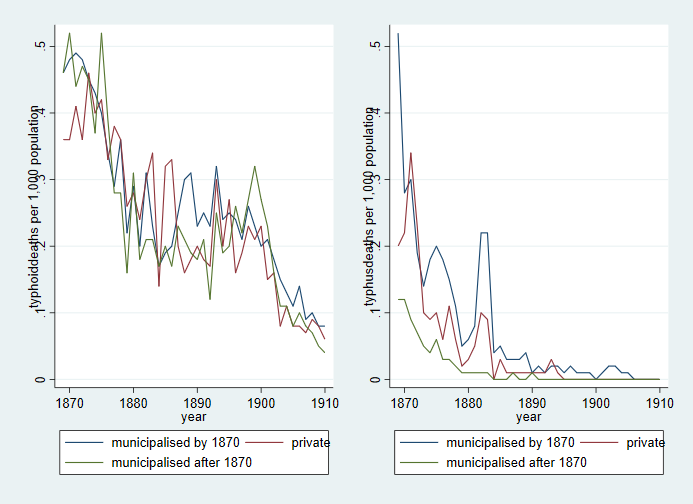

Supplement: Supplementary file 3 — Supporting Information [file EHR-76-624-s002.zip › deposit/output/figures/Figure7.png]

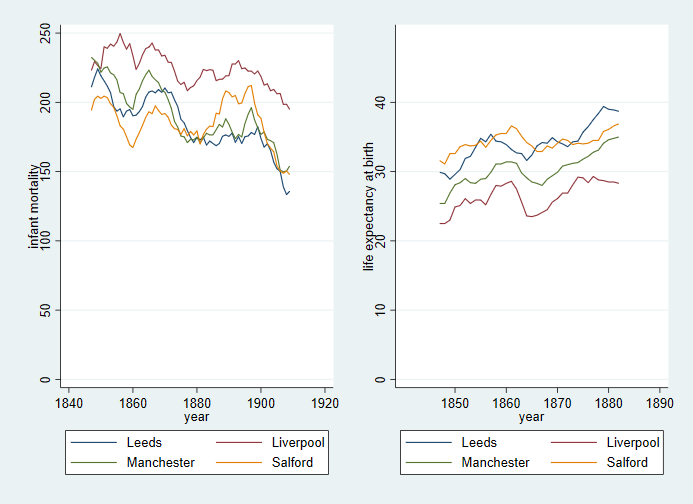

Supplement: Supplementary file 3 — Supporting Information [file EHR-76-624-s002.zip › deposit/output/figures/Figure8.png]
